# Directed Self‐Assembly of Magnetic Bioceramic Deep Inside Dentinal Tubules May Alleviate Dental Hypersensitivity

**DOI:** 10.1002/advs.202507664

**Published:** 2025-07-17

**Authors:** Shanmukh Peddi, Prajwal Hegde, Prannay Reddy, Anaxee Barman, Arnab Barik, Debayan Dasgupta, Ambarish Ghosh

**Affiliations:** ^1^ Centre for Nano Science and Engineering Indian Institute of Science Bangalore 560012 India; ^2^ Centre for Neuroscience Indian Institute of Science Bangalore 560012 India; ^3^ Department of Physics Indian Institute of Science Bangalore 560012 India; ^4^ Theranautilus Private Limited Bangalore 560012 India

**Keywords:** bioactive glass, dental hypersensitivity, magnetic manipulation, nanomedicine, self‐assembly

## Abstract

Delivery of regenerative medicine in complex, microscale topographies can revolutionize multiple areas of healthcare, including but not limited to orthopaedics and dentistry. The technical challenges include navigation and regeneration of nanoscale biosimilars with spatial control, necessitating a different technological approach, as demonstrated here. The specific problem addressed here is dental hypersensitivity, which occurs when dentinal tubules are exposed to the external environment through enamel loss or cementum erosion of the tooth, thus stimulating nerves located in the peripheral odontoblast zone of the pulp. Existing treatments, such as sensitive toothpaste and adhesive resins, are limited to the surface and can only provide short‐term relief. Here, we deploy a confluence of distinct experimental strategies to develop a magnetic bioglass‐based nanomaterial called “CalBots,” consisting of a Calcium‐based colloidal gel that self‐assembles into short chains under optimized conditions of external magnetic fields and interparticle interactions and penetrates more than 300 µm deep inside the complex topography of the dentine tissue. Subsequently, it triggers the formation of a biocompatible seal, thus protecting the exposed tubules and their nerve fibers from external stimuli, for both human and murine teeth. The controlled animal trial shows a full recovery from dental hypersensitivity within the treatment group.

## Graphical abstract

Magnetically guided bioceramic nanoparticles (“CalBots”) achieve deep dentinal tubule occlusion via directed self‐assembly under externally applied magnetic field. Various visualization techniques and a novel mouse behavioral assay indicate that CalBot‐induced plugs may reduce dentinal sensitivity, offering a promising strategy for future dentin hypersensitivity treatments.

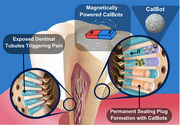

## 1 Introduction

**Figure 1:**
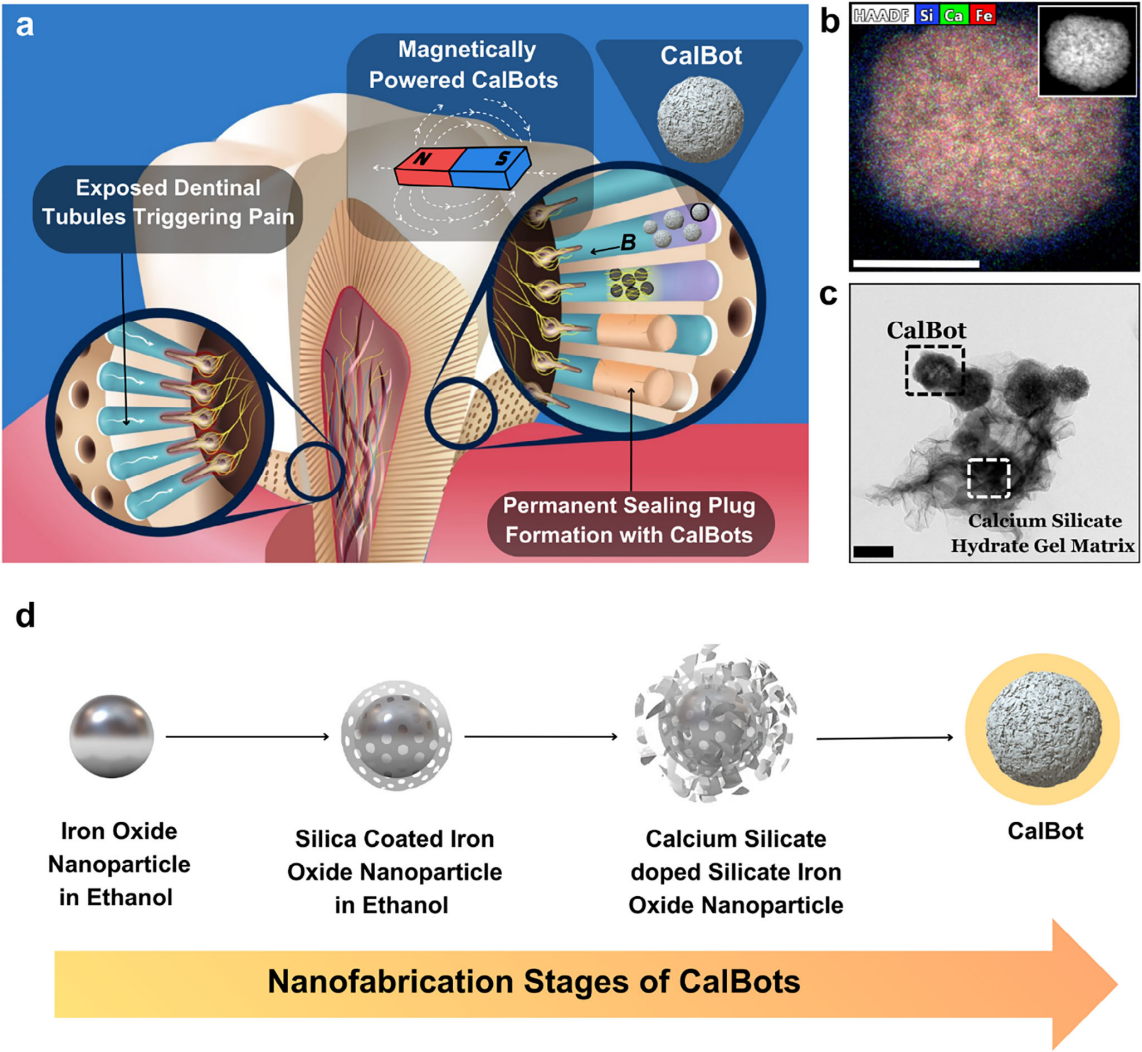
a) Schematic representation of dental hypersensitivity and the physiology of plug formation inside dentinal tubules using CalBots suspended in CaO gel. b) High‐angle annular Dark‐Field (HAADF) image of CalBots with elemental analysis. c) Transmission Electron Microscope (TEM) image of CalBots suspended in CaO gel for 30 days showcasing the formation of cohesive calcium silicate hydrate gel matrix amongst neighbouring CalBots. The scale bar represents 200 nm for both the images. d) Schematic representation of nanofabrication stages of CalBots.

Dentinal hypersensitivity (DH) is a condition that results in sharp pain triggered by routine activities or common substances encountered in daily life, such as tooth brushing, exposure to cold, sugary, or acidic beverages, or cold air. DH is a broad term encompassing a range of clinical presentations. The most common form is linked to cervical non‐carious lesions, often associated with gingival recession^[^ ^1^ ^]^ and the loss of dental cementum due to mechanical abrasion or chemical erosion. Another contributing factor may be the loss of the thin enamel layer at the cervical margin. These changes lead to the exposure of open dentinal tubules to the oral environment, resulting in fluid movement within the tubules and subsequent hypersensitivity. Irrespective of what the underlying cause maybe,^[^ ^2^ ^]^DH is believed to be caused by fluid movement within exposed dentinal tubules due to various stimuli, such as temperature changes, mechanical friction, and osmotic forces, as presented in **Figure** **1**a. This fluid flow within the dentin stimulates nerve endings along the pulp canal, resulting in the sensation of pain, which can range from mild discomfort to severe agony.^[^ ^3^ ^]^ DH is a globally prevalent issue, affecting the quality of life of more than one billion humans.^[^ ^4^, ^5^, ^6^, ^7^, ^8^, ^9^ ^]^

Various interventions have been proposed to alleviate DH, however, the broader strategy towards alleviating DH involves blocking the exposed dentinal tubules and isolating the dentinal fluids from the external environment.^[^ ^10^ ^]^ Laser‐induced occlusion of exposed dentinal tubules is also employed for DH. However, it is reported to be ineffective^[^ ^11^ ^]^ as it potentially denatures superficial dentine through melting and charring, further compromising dentin microstructure. Desensitizing toothpaste and resin sealing agents provide superficial coating for the tubule typically requiring 2–4 weeks for noticeable effects;^[^ ^12^ ^]^ their efficacy wanes without daily application.^[^ ^13^ ^]^ Resin‐based dentin sealers exhibit less than 10% effectiveness, with no sealing effect after four weeks,^[^ ^14^ ^]^ highlighting the need for more enduring DH solutions.

Therefore, the long‐standing technical challenge is to achieve a permanent deeper penetration of the sealant, to shield the pulp from external perturbations. This is especially challenging due to the complex topography, particularly the microscale porosity of the dentine tissue, made of an array of micron‐sized tubules, which has historically limited the depth of penetration for many conventional solutions. While recent advancements relying on slower, passive diffusion have shown promise in achieving deep penetration, such as the use of highly concentrated polyelectrolyte‐calcium suspensions,^[^ ^15^ ^]^ here we present an approach based on active manipulation of magnetic nanoparticles that achieves deeper penetration in a shorter time. As we demonstrate in this paper (see Figure 1a), the solution requires a confluence of novel approaches, specifically: (i) synthesis of a magnetically manoeuvrable bioglass nanomaterial, followed by (ii) devising an optimal active manipulation strategy using magnetic field induced self‐assembly, such as to penetrate deeper than few hundred micrometers (µm) into the dentinal tubules in a relatively short timescale. The final step (iii) requires a controlled self‐setting hydraulic cement reaction, wherein they transform into a cohesive matrix of calcium silicate hydrate gel, which undergoes solidification to yield a robust and enduring sealing plug impervious to external influences. In this work, we have developed an effective means of plugging dentinal tubules which are implicated in DH. Mouse model experiments indicate that this approach is effective in reducing symptoms associated with hypersensitivity.

## 2 Results and Discussion

### 2.1 Nanomaterial Platform

CalBots, as shown in Figure 1b, are approximately spherical core‐shell, superparamagnetic structures with an average diameter of 387 ± 55 nm. Nanofabrication (Figure 1d) and characterization details of CalBots are presented in Sections 1 and 2: Materials and Methods. The inner core is made of iron oxide enclosed by an outer layer composed of calcium silicate embedded in a silica shell, as shown in the TEM analysis of the CalBots in Figure 1b. During our therapy, CalBots were suspended in a gel of 1 mg/ml calcium oxide (CaO) and water, leading to the formation of calcium hydroxide (Ca(OH)_2_). The CalBot suspension, in the presence of ambient CO_2_, transforms into a cohesive matrix of calcium silicate hydrate gel, which undergoes solidification over 2–20 hours to yield a structural matrix imperative for sealing the exposed dentinal tubules.^[^ ^16^, ^17^ ^]^ The mechanism of plug formation for such composites has been studied previously by various groups for a variety of alternate compositions.^[^ ^18^ ^]^ The matrix formation was confirmed by suspending 1 mg of CalBots in 1 ml solution of 1 mg ml^−1^ (w/v) calcium oxide gel along with a control sample containing CalBots suspended in deionized (DI) water. Both the samples were preserved under sterile conditions, following which TEM imaging was performed at different time points to confirm the formation of a calcium silicate hydrate gel matrix in the CalBot – CaO sample (Figure 1c), in contrast to its absence in the control sample for CalBot‐DI water.

### 2.2 Magnetic Manipulation of CalBots for Sealing Dentinal Tubules (In Vitro) and Role of Topography

To gain a quantitative understanding of the penetration of the CalBots under magnetic manipulation, we first consider their dynamics in bulk liquid under a magnetic gradient field. For our experiments, the magnetic field and the direction of the magnetic force have been kept parallel. In agreement with prior work, the CalBots assembled into chains and were pulled by the magnetic gradient field, countered by the hydrodynamic drag. Control experiments performed with CalBots in DI water were used to calculate a single CalBot's velocity and magnetic moment (see Section S1 and Figure S1, Supporting Information).

**Figure 2:**
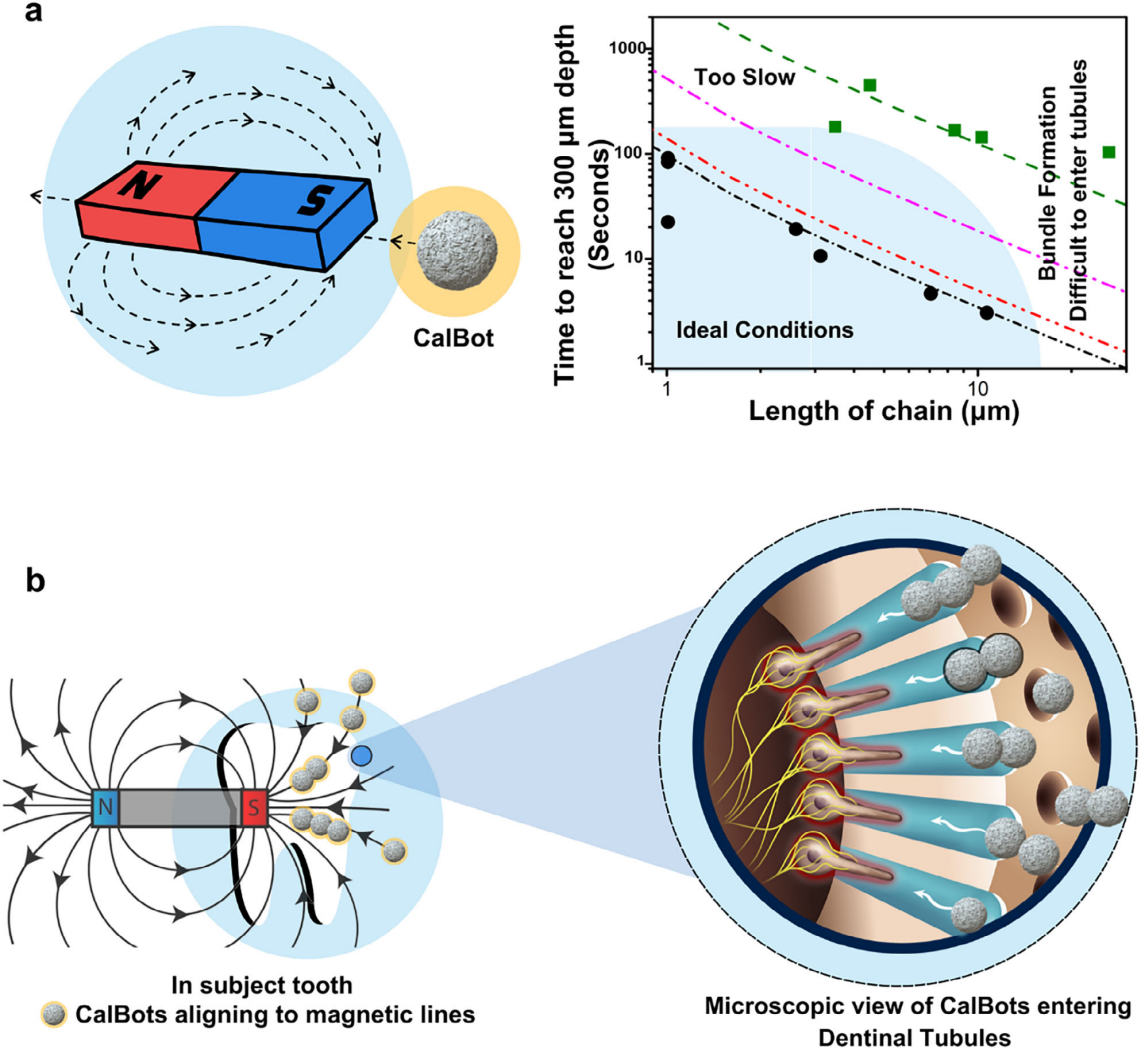
a) Schematic showing field lines generated by a Neodymium magnet. The Graph shows the time taken by a chain of CalBot to reach 300 µm for various chain lengths. The dotted, solid curves are simulated velocity estimations for different gradient magnetic fields: 1500 G/cm (black), 1250G/cm (red), 750 G/cm (pink) and 400G/cm (green). The solid dots represent experimentally observed velocity data converted to time required to reach the depth of 300 µm for 1500 G/cm (black) and 400 G/cm (green) gradient magnetic fields. b) Schematic of CalBot chains moving towards a permanent magnet.

Next, we discuss the subtleties of the experimental design for maximum penetration of the CalBots into the dentinal tubules. The speed of the CalBot chains is directly proportional to the length of chains, implying larger chain lengths are preferable for greater penetration into the dentinal tubules for a given time of operation. As shown by the simulated graphs in **Figure** **2**a, as chain length increases, the time required to reach a depth of 300 µm inside the dentinal tubules reduces, as would be preferred in a clinical setting (see Section S1, Supporting Information for velocity calculation). However, increasing the magnetic field strength would make it energetically favorable to form bundles rather than chains, which will limit their entry inside the tubules. Faraudo et al.^[^ ^19^ ^]^ reported an aggregation parameter N* to determine the structure of field‐induced self‐assembly of superparamagnetic particles into chains. This aggregation parameter is defined as: $N^{*}=\sqrt{\phi_{0}e^{\Gamma -1}}\;$ where $\phi_{0}=\frac{\pi }{6}\;d^{3}n\;$ is the volume fraction of the suspension occupied by CALBOTs, and $\Gamma =\frac{\mu_{0}m^{2}}{2\pi d^{3}k_{B}T}$ is a coupling parameter representing the ratio between the maximum value for the attractive magnetic energy and the thermal energy. In these equations, *n* is the number of CALBOTs per unit volume, *d* is the diameter of CALBOTs, *m* is the magnetic moment, μ_0_ is the magnetic permeability of free space and *k_B_T* is the thermal energy. When 1 < N* < 10 the energy balance favours chain formation. Higher N* causes formations of bundles that may have difficulty entering the dentinal tubule openings. Lower N* would not favour chain formations, and as a result, adequate plug formations would not be observed. For our experiments, N* < 10 could be maintained with a particles per unit volume concentration less than 8  ×  10^21^ for CALBOTs with saturation magnetization, *m*  ≈  2.5–5  ×  10^−14^ *Am* ^2^. With this range of particle density, a sufficient length of chain formation was observed and could be manipulated with reasonable velocity. As indicated in Figure 2a, therefore, careful optimization of experimental parameters is needed to ensure that entry into tubules is neither too slow nor hindered by bundle formation. For all our subsequent experiments, we operated within this regime of CalBot density and magnetic field strength.

To demonstrate the penetration of the CalBots deep into the tubules and subsequent plug formation with sealing ability, we performed experiments with *ex vivo* human teeth samples (Section 3: Materials and Methods). Figure 2b schematic shows how a gradient magnetic field was used to pull the CalBots deep into the tubules for 20 minutes.

**Figure 3:**
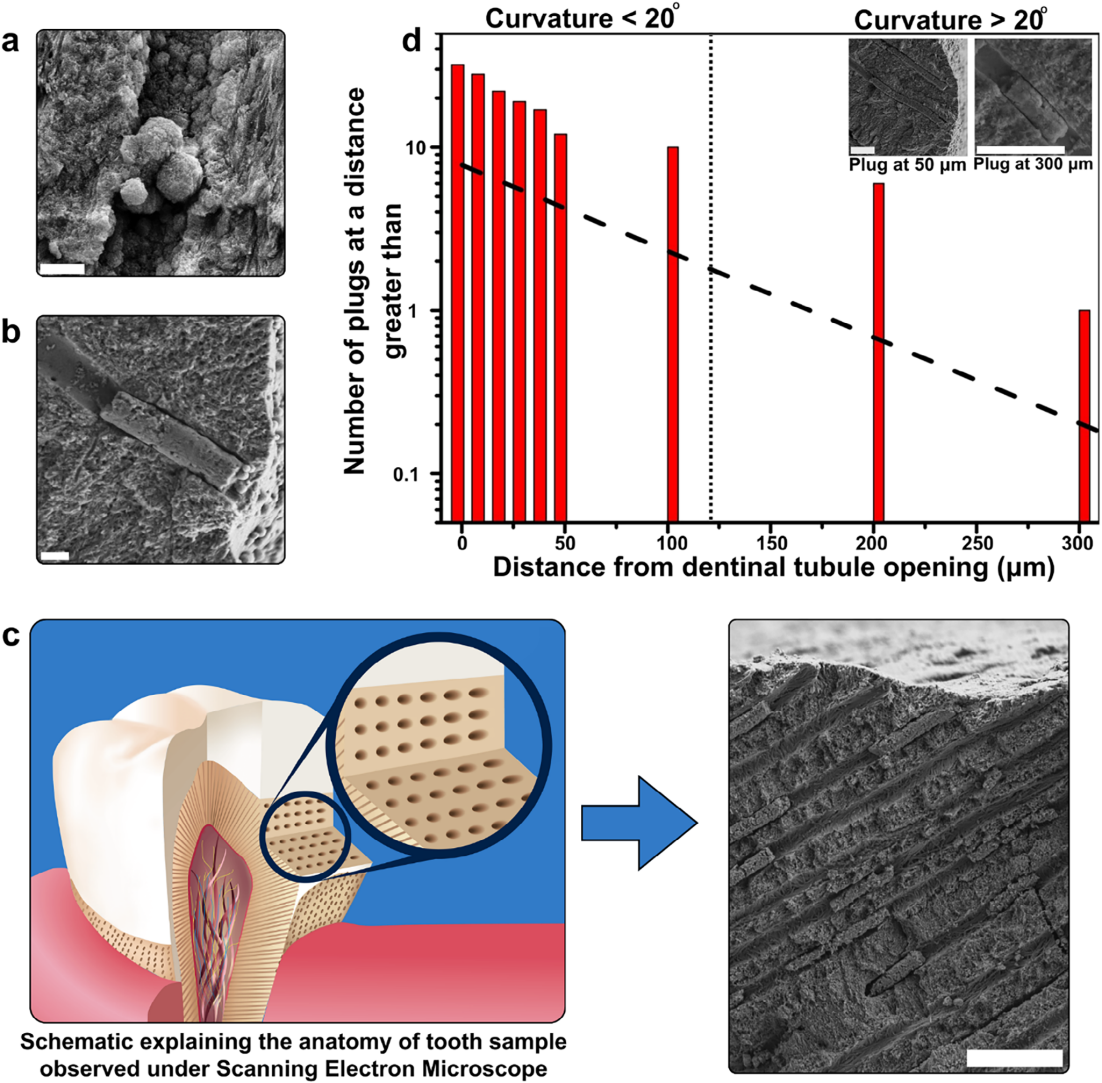
a) Scanning Electron Microscope [SEM] analysis of in vitro human teeth samples treated with CalBots suspended in distilled water presenting weak plugs formation. b) SEM analysis of in vitro human teeth samples treated with CalBots treatment solution giving structurally robust cementing plugs. c) Representative SEM image of multiple robust cement plugs formation within the experimentally exposed cross‐section of dentine tissue d) Cumulative count of CalBot plugs observed from the exposed dentine. Most plugs were observed at a depth between 10 to 100 µm from the edge of the exposed dentine. The inset shows examples of plugs overserved at 50 and 300 µm from the dentin‐enamel junction. The scale bar for (a) is 500 nm, for (b) is 1 µm, for (c) is 10 µm, and for (d) inset the scale bars represent 5 µm for both.

Initial experiments with CalBots suspended in deionized water showed the presence of CalBots deep inside the dentinal tubule. However, as shown in **Figure** **3**a, cement plugs or plug‐like structures were not formed. Following this experiment, the CalBots were placed in 1 mg/ml w/v solution of calcium oxide‐distilled water gel. Examples of plug formation within a dentinal tubule at depths up to approximately 300 µm were observed, as shown in Figure 3b, confirming the importance of CaO in the cementification process. A few plugs were also observed at depths of 400 or 500 µm from the dentinal opening, as shown in Figure S2 (Supporting Information). In the subsequent section, we address the question of the spatial distribution of the depth of plugs. The plugs are designed to increase the flow impedance of the narrow dentinal tubule locally, which in turn could reduce the fluid flow that could trigger hypersensitivity. The structural integrity of the plugs under pressure‐driven air flow was tested to ascertain the air‐tightness of the cement plugs. The results show an 87.7% reduction in dentine permeability post treatment (see Section S2 and Figure S3, Supporting Information). We believe the significant depth of the occlusions may automatically result in enhanced resistance toward mechanical destabilization, as suggested in the existing literature,^[^ ^20^, ^21^ ^]^ which in turn could potentially lead toward a long‐lived therapeutic intervention.

The question of optimal chain length is not only relevant for moving at a reasonable speed but also plays a critical role in achieving maximum penetration into the complex topography of the dentine tissue. Zaslansky et al.^[^ ^22^ ^]^ reported that most tubules do not extend at right angles from the dentine‐enamel junction. Their orientations change within the first half‐millimetre zone beneath the dentin‐enamel junction. This change in the orientation of the tubules introduces topography‐influenced filtering of longer chain lengths that do not reach greater depths. More cement plugs are formed within the first 100 µm of the exposed dentine, and almost all dentinal openings are closed, as seen in Figure 3c. However, as the depth from the dentine enamel junction increased, fewer plugs were observed. As shown in Figure 3d, the distribution of the depths beyond which a plug is observed falls exponentially and is extremely rare beyond ≈200 µm. This can be explained by the topography of the dentine, which prevents longer chains from reaching deeper into the tubules (see Figure S1d, Supporting Information for an example). This suggests that our approach naturally adapts to the varying tubule orientations. The magnetic field guides the CalBots into the tubules, but the complex topography of the dentine tissue acts as a natural filter, allowing shorter chains to penetrate deeper where tubule orientations change.

### 2.3 Spatial Distribution of Occlusion Depth Using Micro‐CT and Image Analysis

Traditional imaging techniques such as scanning electron microscopy (SEM) and confocal microscopy provide qualitative insights into the occlusion of dentinal tubules. However, they do not allow for statistical analysis of the depth of occlusion due to their limited field of view and/or lack of optical penetration into the highly scattering dentine tissue. To address this limitation, we employ a novel micro‐CT‐based method using silver nanoparticles as contrast agents. This technique enables a quantitative evaluation of tubule occlusion depth, demonstrating how CalBots enhance dentine occlusion more effectively.

### 2.4 Micro‐CT Imaging and Silver Nanoparticle Contrast

**Figure 4:**
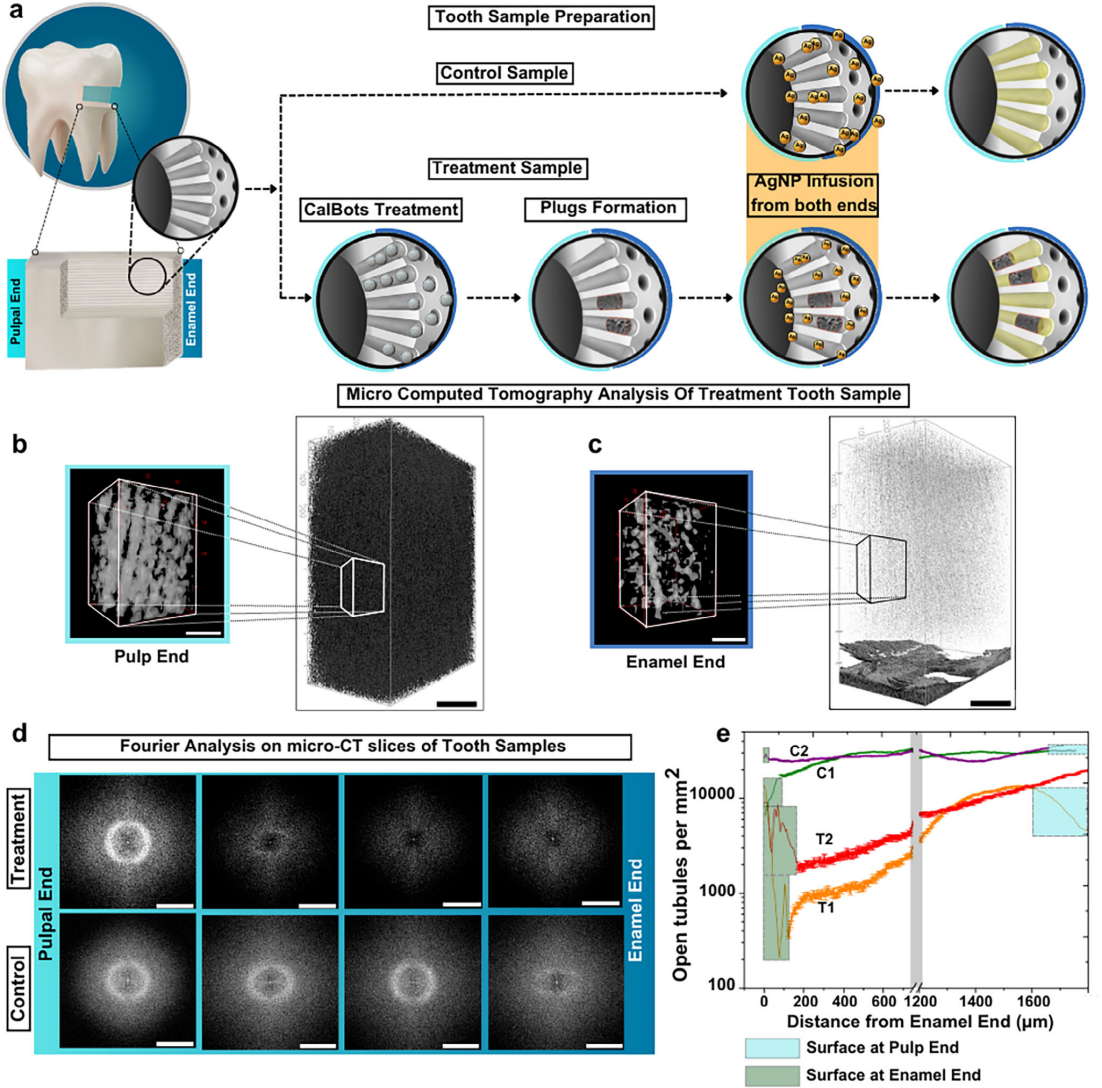
a) Schematic representation showing the method of sample preparation for micro‐CT. For the control samples, the dentine discs were dipped in silver nanoparticle solutions, so that the nanoparticles can enter the tubules from either ends. For the treatment samples, a magnetic drive was used to achieve occlusion in the depths of the tubules before dipping them in the silver nanoparticle solutions. b) 3D reconstruction of micro‐CT stacks to show the open tubules in a CalBot‐treated sample near the pulp end (PE). The scale bar on the complete reconstruction is 100 µm. The scale bar on the zoomed‐in view is 10 µm. c) 3D reconstruction of the micro‐CT stacks near the Enamel End (EE) of the CalBot treated sample showing marked reduction in silver contrast. The scale bar on the complete reconstruction is 100 µm. The scale bar on the zoomed‐in view is 10 µm. d) Snapshots of Fourier transform of select slices from the micro‐CT data showing changes in regularity and gradual disappearance of open dentine contrast from PE to EE where treatment was administered. Such marked changes are not observed for the control sample. The scale bar for Fourier analysis is 0.5 cycles/ µm. e) The plot of open tubules where silver nanoparticles could reach to provide CT contrast as a function of distance from the Enamel End. Plots labelled C1 and C2 represent two control samples and T1 and T2 represent samples where CalBots have been driven in from their enamel ends.

Micro‐CT imaging was employed to visualize and quantify the extent of Calbot penetration and occlusion within dentinal tubules. Following Calbot treatment, the dentine samples were immersed in a silver nanoparticle solution containing 40 nm particles for three days to ensure thorough infiltration into open tubules (**Figure** **4**a). For control, the samples were directly immersed in silver nanoparticle solution. Open tubules got selectively filled with silver nanoparticles, while occluded tubules remained unmarked due to the calcium deposits, which do not provide CT contrast against dentine. To elaborate, there was no contrast from certain regions inside the tubules, which were out of reach for the silver nanoparticles, defined by the region between the shallowest and the deepest plugs. This differentiation allowed for a clear distinction between open and occluded tubules, offering an accurate method to measure the depth of occlusion.

The high‐contrast imaging enabled the 3D reconstruction of tubule structures and allowed for threshold‐based quantification of open tubules. As shown in Figure 4b,c for a CalBot treated sample, there is a marked difference between open tubules where silver nanoparticles could reach to provide CT contrast and occluded tubules near the enamel end where due to treatment and plug formations silver nanoparticles could not enter. The qualitative changes in the contrast of the two ends of the dentine discs is in line with our earlier observations of the occlusion depth of 300–500 µm.

### 2.5 Quantitative Analysis of Tubule Occlusion

Using ImageJ and a particle counting algorithm, the number of open tubules per square millimetre was computed (see Materials and Methods: Section 3 (Micro‐CT analysis and FFT analysis for details). The processed micro‐CT data showed a marked reduction in open tubules near the enamel end. In contrast, untreated samples exhibited a relatively uniform distribution of open tubules throughout the depth of the dentine (see Figure 4e). We also shade the regions near the enamel and the pulp ends, where the tubule counts became unreliable. This was primarily due to the intrinsic roughness of the sectioned surface, as well as the nanoparticles that entered the tubules from the enamel end. An example of the rough surface can be clearly seen in the lower part of Figure 4c. These regions were slightly different for each sample, as would be expected, and therefore marked in Figure 4e and were not considered hereafter.

Our study examines how dentine tubule density changes from the enamel end to the pulp end, in alignment with findings from previous morphometric analyses. Specifically, the control samples exhibit a similar trend as reported in the literature^[^ ^23^ ^]^ where the tubule density decreases toward the enamel, maximum by a factor of two. However, our treatment samples showed a pronounced reduction in the number of open tubules due to Calbots' occlusion effect. As a quantitative measure, we find the number of open tubules for samples T1 and T2 was ≈13 000 per mm^2^ and ≈20 000 per mm^2^, respectively, near the pulp end. These numbers reduced to ≈1200 per mm^2^ and ≈3000 per mm^2^ respectively, at 500 µm depth from the enamel end, corresponding to 90% and 85% of the tubules having occlusions at such great depths.

The findings were further corroborated through Fast Fourier Transform (FFT) analysis, which confirmed spatial frequency variations corresponding to decreasing open tubule density. Fourier transform, as a method of analysis, has been used to study nanopore arrangements ^[^ ^24^, ^25^, ^26^ ^]^. Qualitatively, as arrangement becomes less ordered, the 2D FFT becomes more blurred.

The 3D reconstructions of micro‐CT stacks visually illustrate this trend, reinforcing the quantitative findings (see the Movie of the 3D reconstruction (Movie S2, Supporting Information)). The circular fringe in the FFT plot, as shown in Figure 4d, represents the presence of dentinal tubules in a disordered arrangement. As more tubules are plugged due to CalBot treatment near the enamel end, the contrast from open dentinal tubules reduces as we approach the enamel end.

### 2.6 Suitability for In Vivo Applications: Animal Toxicity Studies

The primary rationale for choosing Generally Recognized as Safe (GRAS) materials for CalBots is to prioritize biocompatibility, aligning with our goal to translate research into clinical trials. Before animal trials, we assessed the toxicity of CalBots in twenty female BALB/c mice aged five weeks (see Section S3, Supporting Information). Grouped according to Table S1 in Supporting Information Section S3 (Supporting Information), mice underwent intraperitoneal CalBots doses (17.5 mg kg^−1^ to 550 mg kg^−1^) following OECD guideline 425. After a 14‐day observation, blood analysis and histopathological examination (see Section S4, Supporting Information) revealed no mortality or distress signs. Body weight, Total Blood Count (TBC), and serum values aligned with the control group (see Section S3 and Table S2, Supporting Information). Results suggest CalBots, up to 550 mg kg^−1^, are safe for mice, indicating potential safety for broader applications.

### 2.7 Efficacy of CalBots in Mice

DH can be a recurring condition, and demonstrating long‐term efficacy requires extended studies in both animal models and human trials. Mice teeth are a reliable marker for evoking spontaneous pain, inducing allodynia.^[^ ^27^ ^]^ To assess DH in mice, we developed a behavioral assay adapted from established two‐bottle preference tests commonly used in taste and temperature preference studies in rodents.^[^ ^28^, ^29^, ^30^, ^31^ ^]^ In these protocols, mice preferentially consume liquids they find more palatable while avoiding aversive stimuli. We modified this paradigm to longitudinally monitor changes in cold water avoidance behavior following enamel damage, as a behavioral correlate of DH. To our knowledge, this represents the first application of a temperature preference‐based assay for assessing dentinal sensitivity in a murine model.

**Figure 5:**
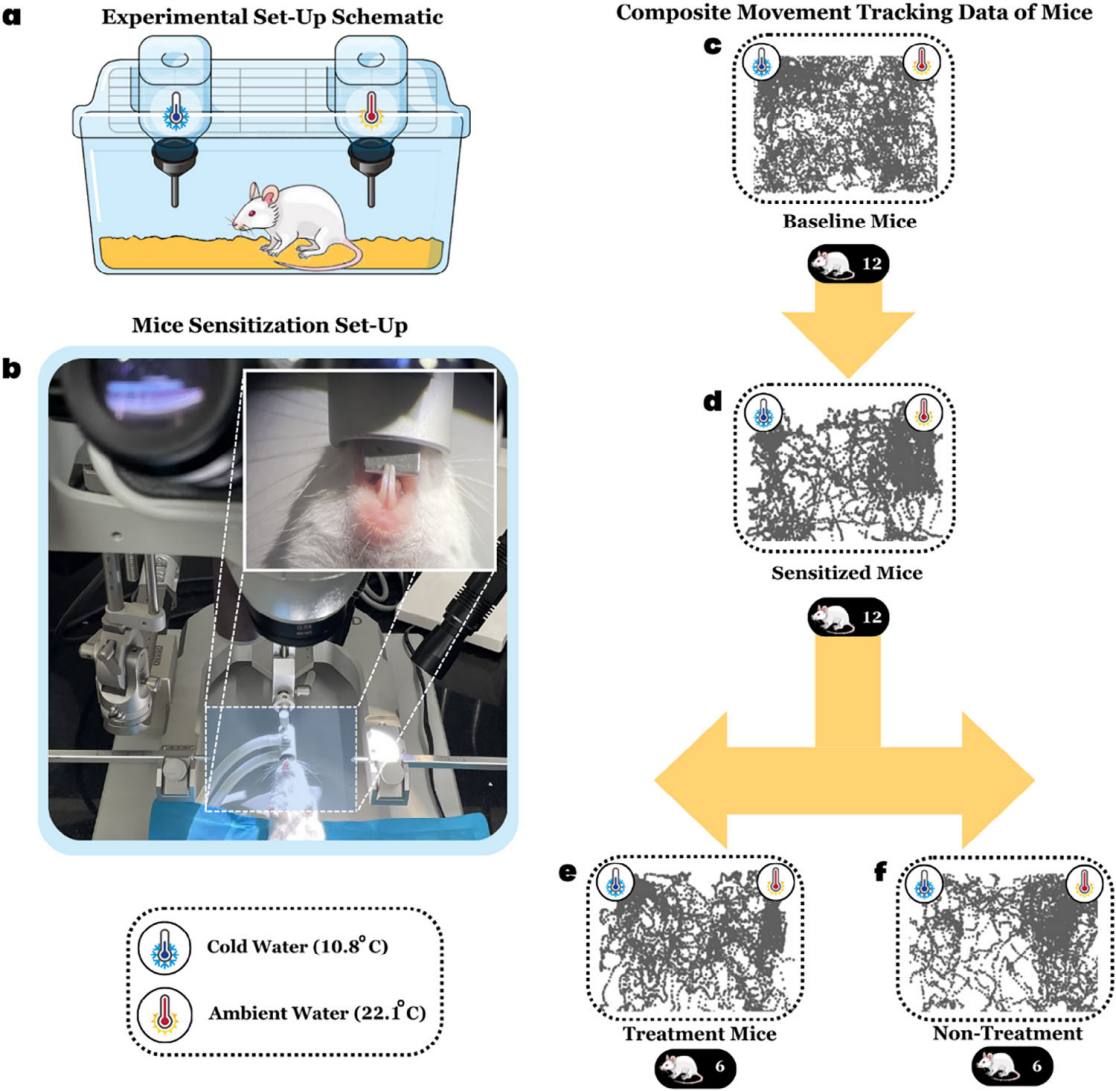
a) A schematic of the experimental setup used to test the preference (if any) of mice for water of either cold or ambient temperature during the three phases of the study (Baseline, Sensitivity, and Treatment). The phases last for seven days each in the following order: Baseline phase, induction of sensitivity, Sensitivity phase, administration of treatment/formulation of the treatment/no treatment for control mice, and Treatment/No‐Treatment phase. b) The experimental setup used for the induction of sensitivity amongst the test mice. A representative image of the tracking of mice movement using the AI software DeepLabCut during a trial from the first day of the baseline phase, c) Baseline phase, d) Sensitization Phase e) Treatment administration group f) No‐treatment administration group.

We observed changes in their water temperature preference to gauge dental hypersensitivity and the effectiveness of enamel damage as a model. We conducted preference tests (see Section 4: Materials and Methods) on healthy mice and those with dental hypersensitivity as illustrated in **Figure** **5**a, before and after treatment with CalBots.

The mice underwent seven‐day baseline trials, ensuring unbiased temperature preferences. DH was induced (Figure 5b,d), and a one‐day recovery succeeded it. DH trials were conducted for seven days to monitor water temperature preference changes. Post‐DH trials, preferences were re‐evaluated for both treated and untreated cohorts. In the treatment cohort, following the administration of CalBots, there was a period of six hours with no water, followed by water consumption for the remainder of the day. Subsequently, there is a 24‐hour no‐water consumption period to prepare the mice for experiment day to understand the treatment cohort's preference towards cold or ambient water. Non‐treatment mice served as additional controls to check for the longevity of the sensitivity caused. AI‐based DeepLabCut© software was deployed to avoid human bias, facilitating nose coordinate tracking, and determining water temperature preferences (see Movie S1, Supporting Information). During baseline trials, the mice exhibited no temperature preference as shown by the tracks in Figure 5c. Following sensitivity induction, clustering towards ambient‐temperature water as shown in Figure 5d indicated cold water avoidance, which persisted in untreated mice throughout the week‐long study (see Figure 5f). CalBot‐treated mice, however, displayed point clustering near both syringes (see Figure 5e), akin to baseline trials, demonstrating reduced cold‐water avoidance. Compared to the baseline, the enhanced clustering in treated mice suggests environmental familiarity. Tracking data explicitly affirmed CalBot's efficacy in diminishing cold water aversion without altering baseline preferences.

To understand the efficacy of CalBot in treating DH, we benchmarked our treatment over four parameters: (i) the Lick Ratio: measuring how many times mice licked cold water compared to ambient water in different stages of the study, (ii) the Bout Ratio: measuring the duration of bouts from every time the mice licked cold water to ambient water during, (iii) the Bout Duration/Lick: measuring the total duration of licking bouts for cold water and (iv) the Lick Number: measuring the total number of times the mice licked cold water in a trial during all three phases of the study. These parameters were observed for 42 trials for each stage of the study.

**Figure 6:**
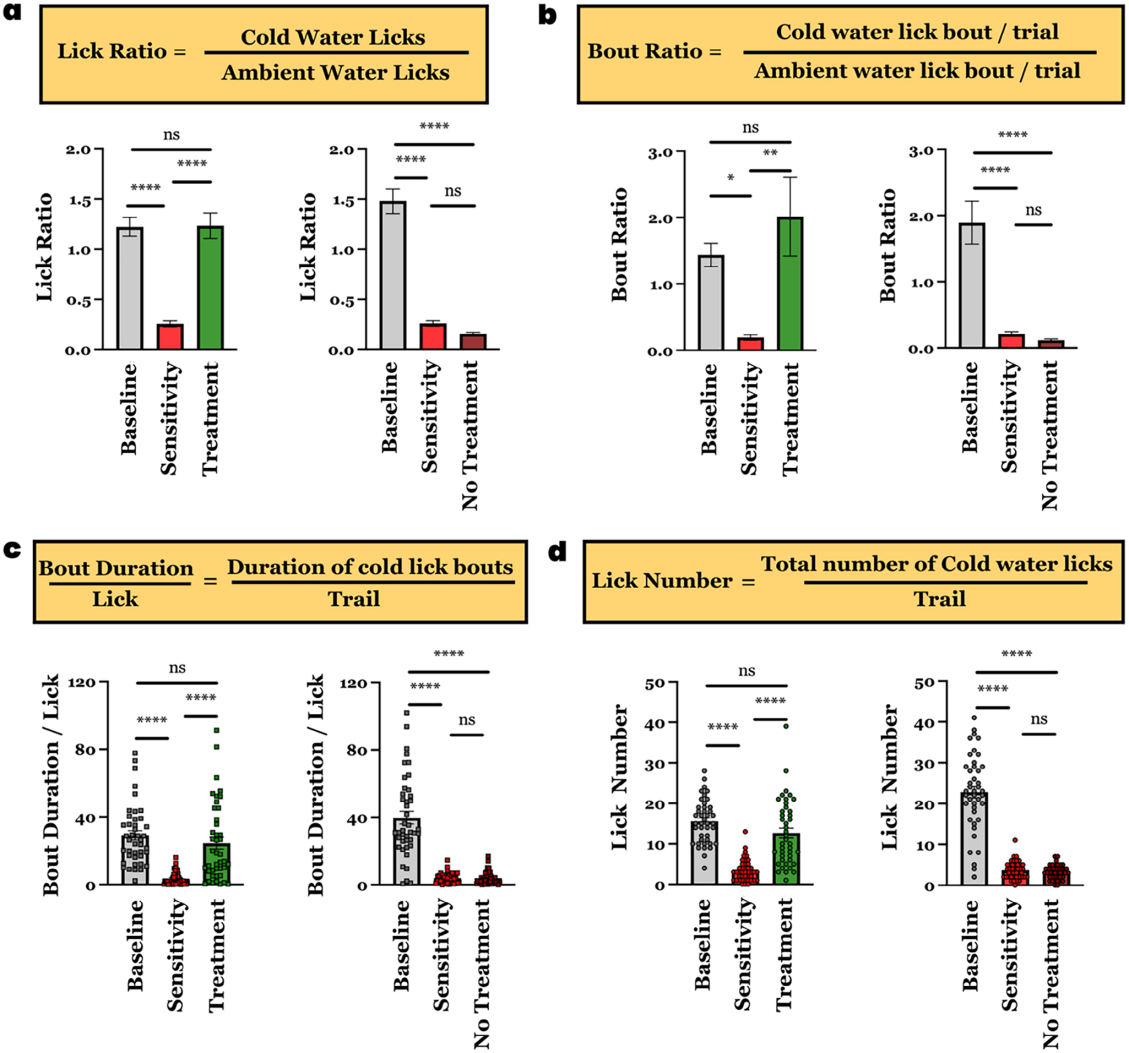
a) Lick Ratio of mice cohort where treatment was administered (Left), compared to untreated mice cohort (Right) b) Bout Ratio of mice cohort where treatment was administered (Left), compared to untreated mice cohort (Right) c) Bout Duration/Lick of mice cohort where treatment was administered (Left), compared to untreated mice cohort (Right). d) Lick Number of mice cohort where treatment was administered (Left), compared to untreated mice cohort (Right). For all experiments significance was established by One‐way ANOVA, ****P value < 0.0001. (n = 6).

As seen in **Figure** **6**a (left), the lick ratio indicates strong avoidance of cold water for mice with hypersensitivity (0.25 with sensitivity against 1.22 baseline). After treatment with CalBots, a strong recovery in the Lick ratio is observed (1.23 post‐treatment). Comparing this data to the untreated mice (Figure 6a, right) the Lick ratio falls to 0.16 with no treatment, clearly showing our intervention's efficacy.

The bout ratio, as shown in Figure 6b, demonstrates that the duration of bouts from every time the mice licked cold water also changes due to sensitivity compared to the duration of bouts for drinking ambient water. This parameter, too, recovers dramatically when treatment is administered.

The Bout duration/lick, as shown in Figure 6c, provides a fascinating insight into the behavioral changes brought about by DH. With the introduction of sensitivity, the mice licked the cold water only for 3.5 seconds on the average, an order of magnitude less when compared to the baseline behavior of 29.2 seconds. In untreated mice, this time does not recover, but once CalBot treatment is administered, the duration shows a dramatic recovery, back to average of 24.5 seconds. The mice also show general behavioral changes, where it avoids cold water altogether, as documented by the Lick number in Figure 6d.

**Figure 7:**
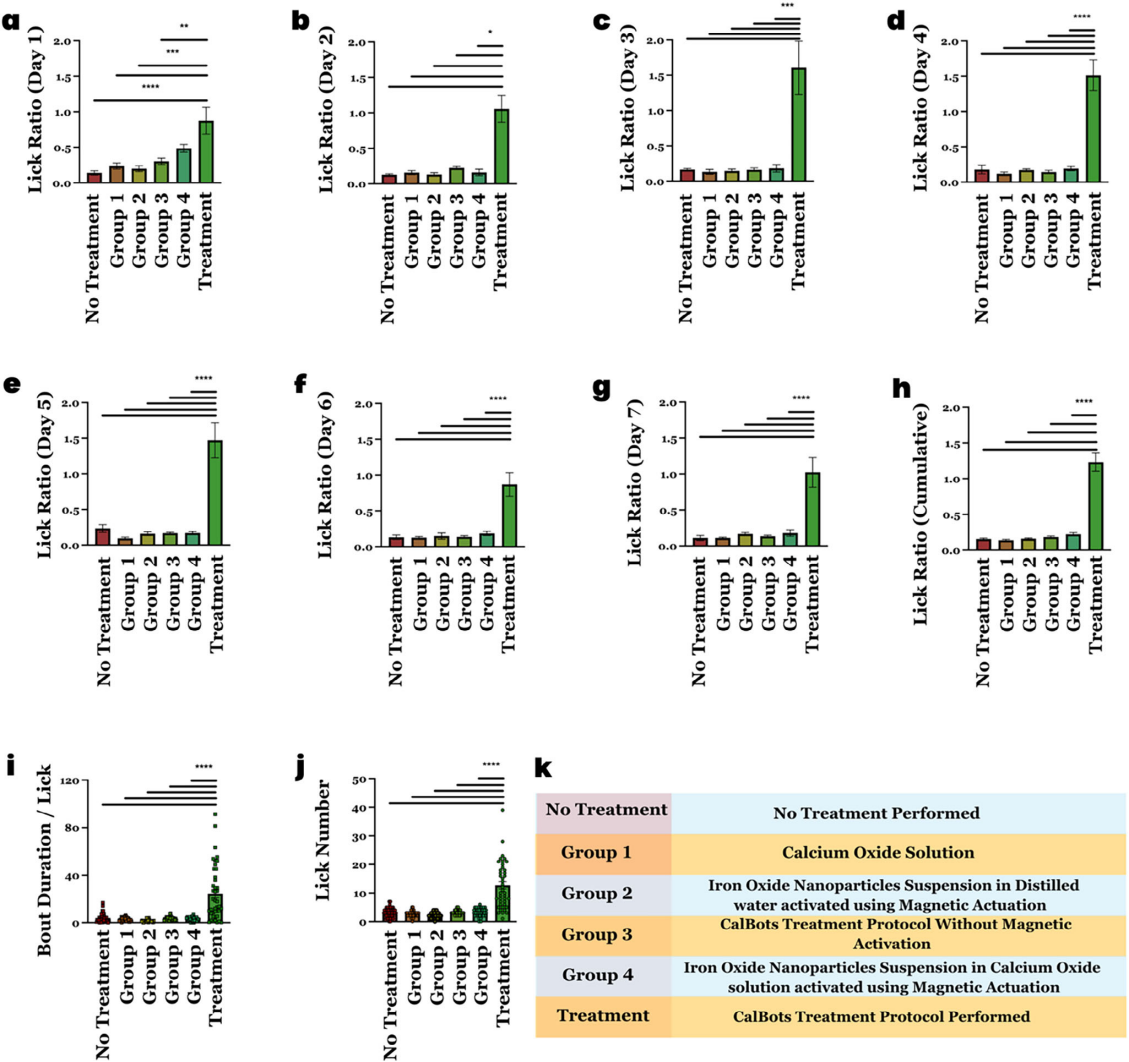
a‐ g) The Lick Ratio of mice in different control groups and the treatment group was observed for 7 days. h) The cumulative Lick ratio after the completion of the experiment. The mean ratio for mice group without treatment, Group 1, Group 2, Group 3, Group 4 and the actual treatment group were 0.16, 0.14, 0.16, 0.18, 0.22, and 1.23, respectively. i) Bout duration/lick for all the groups and j) Lick number. k) Table representing the different groups of treatments mice received. The cohort size for the groups were Group 1 (n = 5), Group 2 (n = 5), Group 3 (n = 5), Group 4 (n = 5) and the actual treatment group (n = 6). For all experiments significance was established by One‐way ANOVA, ****P value < 0.0001.

The data in Figure 6, represents end points after the experiments were concluded. Now we discuss the temporal implications of CalBot treatment and the dramatic improvement offered by CalBots compared to all existing ideas for treating dental hypersensitivity. We plotted the comparative Lick Ratios for the mice groups for seven consecutive days (**Figure** **7**a–g). The cumulative Lick Ratios, bout duration per lick and number of licks as shown in Figure 7h–j. Interestingly, for Groups 1 and 3, where only some form of a cementing layer was administered without any magnetic actuation, the Lick Ratio improved on Day 1 compared to no treatment (Figure 7a). This mode of treatment is analogous to the current state of the art, where sensitive toothpaste forms a protective layer over the exposed dentine. As evidenced by all previous reports, this temporary coating gives inadequate relief even on the day of the treatment and provides no benefit in the long run. Groups 2 and 4 use magnetic actuation of nanoparticles and demonstrate that even partial occlusion of exposed dentin can alter behaviors in mice that are consistent with those associated with dental hypersensitivity However, all of the treatment modalities in the control groups show poor efficacy in providing pain relief over a long time compared to our cement formation method deep inside the dentine using CalBots. Without cementing plug formation, removal of the magnetic field dispersed the agglomeration, resulting in DH relapse within a day.

Based on our animal experiments for the treatment cohort, it took a mere 6‐hour window for CalBots to form plugs within dentinal tubules, which represents a possible treatment strategy to potentially alleviate DH. Our behavioral observations suggest the recovery behavior due to CalBot plug formation persists for at least two weeks post‐induction in mice. From a mechanistic standpoint, we believe that the plugs formed by CalBots inside the dentinal tubules can last long, because these blockages are shielded from the daily abrasive forces faced by the surface of teeth. This hypothesis stems from previous studies^[^ ^32^, ^33^ ^]^ where calcium trisilicate was shown to persist after one year when subjected to the dentine‐pulp response.

In a potential clinical scenario, this treatment may possibly involve a suspension of CalBots applied to exposed dentin surfaces, guided by a carefully designed handheld or intraoral magnetic applicator. As demonstrated in our ex vivo experiments, magnetic actuation from a single accessible surface is sufficient for effective tubule penetration, with treatment durations expected to range between 10–20 minutes. Future translational studies will focus on optimizing magnetic delivery systems and streamlining procedural workflows.

## 3 Conclusion

This study presents an effective method of blocking exposed dentinal tubules which may be promising for managing dental hypersensitivity. Achieving deep penetration into the dentinal tubules, which are highly complex and characterized by their microscale porosity, required a delicate balance of forces for collection of nanoparticles intended for penetration into exposed tubules. The magnetic drive induces the self‐assembly of CalBots into chain‐like structures, while the physics of interparticle interactions ensures that these chains remain flexible enough to navigate through the intricate and variable geometry of the tubules. The ability of CalBots to form cement‐like plugs further demonstrates the sophistication of this system, as the plugs undergo solidification to create a robust, biocompatible seal deep within the tissue. It required careful optimization of several parameters, including magnetic field strength, chain length, and the concentration of CalBots to ensure that the colloidal particles not only reach the desired depth but also self‐assemble into stable structures capable of occluding the dentinal tubules. This balance is essential, as overly strong magnetic fields could result in bundling of particles, preventing effective penetration, while weak fields would limit the formation of useful chains. Thus, this method goes beyond simply pulling a few particles with a magnet; it involves the precise engineering of dynamic self‐assembly at a microscale level, which has rarely, if ever, been applied in this context. Indeed, there have been multiple approaches to using maneuverable nanostructures, also referred to as nanorobots or nanomotors,^[^ ^34^, ^35^ ^]^ for therapeutic applications, where the structures were manipulated in human *ex vivo* organs, including human blood^[^ ^36^ ^]^ cancer microenvironment^[^ ^37^ ^]^ and teeth^[^ ^38^ ^]^ as well under animal in vivo conditions such as porcine eyes^[^ ^39^ ^]^ gastric cavity^[^ ^40^ ^]^ intraperitoneal cavity^[^ ^41^ ^]^ and urinary bladder^[^ ^42^ ^]^ of mice using remote energy sources like magnetic field, acoustic and chemical propulsion. These precisely targeted drug delivery approaches are promising yet distinct from our demonstration of targeted regenerative medicine. Interestingly, while medical professionals routinely use calcium silicate‐based cement due to their high biocompatibility and osteoconductivity^[^ ^43^ ^]^ for example, in applications such as orthopedic spinal fusion surgeries,^[^ ^44^ ^]^ and regenerative dentistry^[^ ^45^ ^]^ as far as we know, this safe, biocompatible^[^ ^46^ ^]^ material platform has never been integrated with magnetic manipulation. Accordingly, this work can form the basis exploring other applications pertaining to degenerative losses in calcified tissue. Additionally, this technique might offer added benefits beyond the relief of dental hypersensitivity. The formation of robust, localized plugs within the dentinal tubules by the CalBots could serve as a physical barrier, potentially preventing bacterial infiltration into the deeper layers of the tooth structure. By reducing or limiting access to the dentinal tubules, which are known pathways for microbial invasion, Although the primary focus of this study was the reduction of fluid flow to alleviate hypersensitivity, the impermeable nature of the plugs formed by CalBots suggests a secondary benefit of minimizing bacterial penetration. This additional function could extend the potential utility of CalBot treatment to not only manage hypersensitivity but also enhance the overall protective barrier of the teeth, promoting long‐term dental health. The CalBot plugs may provide an extra layer of defense against bacterial infection as in early dentinal caries. This also could be particularly valuable in protecting the sensitive pulp tissue from other deleterious insults to tooth (cervical abrasions, erosions); congenital dental anomalies (amelogenesis imperfecta) and iatrogenic causes (dentinal perforations during root canal procedures). While CalBots exhibit deep penetration for tubule occlusion, future studies will focus on evaluating long‐term outcomes under real‐world conditions. Indeed, the ideas and demonstrations in this paper are similar in spirit to Feynman's tiny mechanical surgeons and the dream of “*small machines [which] might be permanently incorporated in the body to assist some inadequately‐ functioning organ*.”^[^ ^47^ ^]^ Last, our comprehensive toxicity evaluations establish that CalBots exhibit a remarkable level of non‐toxicity, emphasizing their safety profile for potential clinical applications.

## 4 Experimental Section

### Section 1: Fabrication of CalBots

The CalBots were fabricated by employing solvothermal synthesis.

Chemical reagents (FeCl_3_. 6H_2_O), Sodium citrate dihydrate (Na_3_C_6_H_5_O_7_. 2H_2_O), Anhydrous Sodium acetate (CH_3_COONa), Ethylene Glycol (HOCH_2_CH_2_OH), Ammonium Hydroxide (NH_4_OH), Tetraethyl orthosilicate (Si(OC_2_H_5_)_4_), Calcium nitrate tetrahydrate (Ca(NO_3_)_2_. 4H_2_O), Calcium oxide (CaO) all chemicals were procured from Sigma Aldrich.

### Synthesis of Iron Oxide Nanoparticles

20 mL of Ethylene glycol was taken in a Teflon tube. 0.2 g of Sodium citrate dihydrate was added to the solution, then 1.2 g of sodium citrate acetate. Finally, 1 g of Iron chloride hexahydrate was added to the solution. The Teflon tube was placed in the autoclave chamber and kept in the hot air oven at 210 °C for 10–12 hr. After this, the sample was cleaned and suspended in ethanol.

### Synthesis of Silica Coated Iron Oxide Nanoparticles

Iron oxide nanoparticles were suspended in an 85 mL ethanol solution.7 mL ammonium hydroxide was added, followed by 0.2 mL TEOS. The solution was placed under sonication for 90 min. At the end of 90 minutes, the particles were cleaned and suspended in 10 mL ethanol.

### Introducing Calcium Silicate on Silica‐Coated Iron Oxide Nanoparticles

About 0.2 – 0.4 g of calcium nitrate tetrahydrate was added to a 10 mL silica‐coated iron oxide nanoparticles ethanol solution. The particle was magnetically separated from the solution and placed in an oven at 40 °C for drying. The sample was annealed for 2–5 hr at 600 °C in an ambient atmosphere. The final product was called CalBot.

### Final Formulation

5 mg of calcium oxide was added to 50 mL of DI water. 0.5 mg of CalBot was suspended in a 2 mL aliquot of Calcium oxide solution.

### Section 2: Characterization of CalBots

Comprehensive characterizations of CalBots was performed to establish its physiochemical properties. Additional characterization of CalBots as it goes through the steps of synthesis shown in Figure 1 can be found in Figure S5 of the Supporting Information. The reactions were described that were believed to be occurring based on the subsequent XRD studies done at different stages of the Calbot formation and Calbot‐CaO solution reaction.

### Transmission Energy Microscopy (TEM) and Energy Dispersive X‐Ray Spectroscopy (EDS) Data Acquisition of CalBots

**Figure 8:**
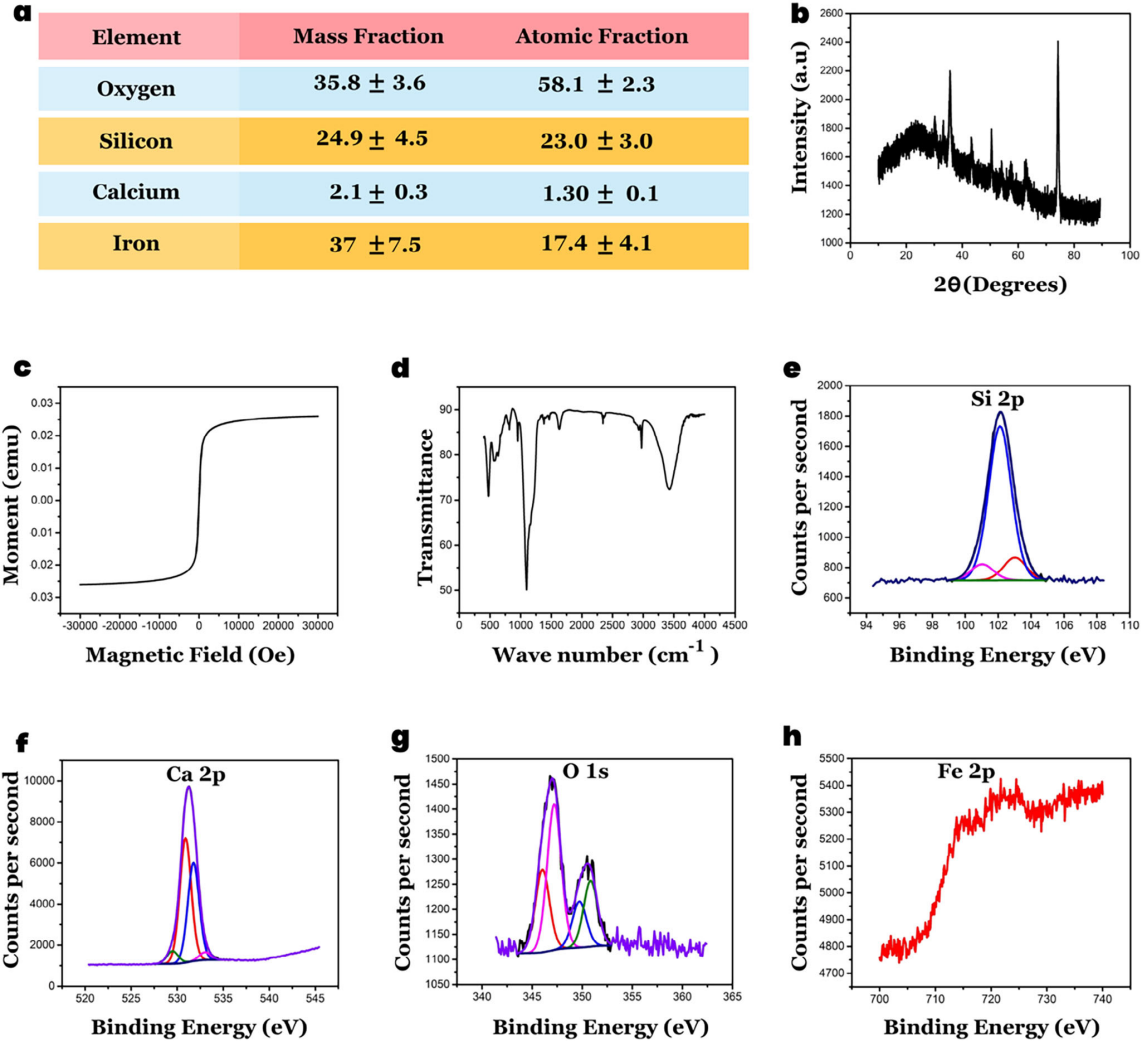
a) Energy Dispersive X‐Ray Spectroscopy (EDS) Data Acquisition of CalBots b) X‐Ray Diffraction (XRD) Characterization of CalBots c) Vibrating Sample Magnetometer (VSM) data analysis of the CalBots. d) Fourier transform Infrared characterization (FTIR) of CalBots. X‐ray Photoelectron Spectroscopy (XPS) characterization of CalBots with peaks observed for e) Silicon, f) Calcium, g) Oxygen, and h) the peaks were absent for Iron due to the effective penetration depth of XPS is less than 10 nm and in case of CalBots Silica and Calcium silicate acts as a barrier.

CalBot powder suspended in Deionized (DI) water was drop‐cast on gold coated TEM carbon grid. The sample was dried under an Infrared lamp and desiccated to avoid moisture formation. Titan Themis 300 KV from Thermo Scientific was used to obtain high‐resolution TEM images. 300 KV acceleration voltage was used for analysis. Super‐X quad EDS detector was used for elemental analysis. As seen in the TEM image, a core Iron oxide nanoparticle of ≈250 nm diameter on which Silica and Calcium silicate deposition was performed, bringing the final size to ≈350 nm. As presented in **Figure** **8**a, EDS information indicates the presence and composition of four elements, i.e., iron, silicon oxide, and calcium. Additional TEM images can be found in Figure S6 in the Supporting Information. The thickness of the shell of the CalBot particles was found to be 34.7 (±) 10.6 nm.

### X‐ray Diffraction (XRD) Characterization of CalBots

The CalBot sample was drop cast on Silicon wafer XRD (Rigaku Smartlab) analysis with parallel beam glazing angle configuration by fixing omega value at 3 degrees. This configuration helped us to capture the signal only from the sample, thereby avoiding the signal from the Silicon wafer. Figure 8b presents the theta ranges from (10°‐80°). Except for the Calcium silicate peak at 33°, all other peaks correspond to Magnetite, which was the chemical configuration of Iron oxide nanoparticles. A detailed analysis of the XRD data for different stages of the plug formation can be found in Figure S5 (Supporting Information)and accompanying comments in Supporting Information.

### Vibrating Sample Magnetometer (VSM) Characterization of CalBots

The magnetic moment of the CalBot sample was quantified by employing a vibrating sample magnetometer (VSM) characterization using Quantum Design's PPMS Versalab. 1 mg of CalBot sample (powder) was filled in an airtight VSM capsule, and the experiment was performed at room temperature, where the applied magnetic field ranges from 30 000 Oe to 30 000 Oe as presented in Figure 8c magnetic moment of the CalBot sample saturated at ∼20 000 Oe which infers that the magnetic properties exhibited by CalBot particles is in a superparamagnetic state.

### Fourier Transform Infrared (FTIR) Analysis of the CalBots Sample

CalBot powder was mixed with Barium sulfate and turned into a pellet for FTIR scan. The Transmittance was plotted against wave number, as presented in Figure 8d

### Zeta Potential and Dynamic Light Scattering (DLS) Characterization of CalBots

Zeta potential and DLS analysis were performed on Zeta PALS (Malvern Panalytical). The surface charge value of CalBot suspended in water was ‐36.70 ± 4.00 mV, and the particle diameter was 387 (±) 55 nm. The polydispersity index of the Calbots consistently falls within the range of 0.055 to 0.15, indicating a reasonably monodisperse particle size distribution.

### X‐ray Photoelectron Spectroscopy (XPS) Characterization of CalBots

CalBot powder, suspended in Deionized water, was drop‐casted onto a copper substrate, and the sample was characterized with XPS (Kratos Axis Ultra). Binding energy range provided for Silicon (95–108 eV), calcium (340–370 eV), Iron (700–740 eV), and Oxygen (520–545 eV). Peak positions were calibrated with respect to adventitious Carbon peak C1s at 284.6. From the XPS data, peaks were observed for Silicon, Calcium, and Oxygen, as represented in Figure 8e–g), respectively. However, we can observe an absence of peak for Iron (Figure 8h). This was because the effective penetration depth of XPS was less than 10 nm, and in the case of CalBots, Silica and Calcium silicate act as a barrier. The remaining peaks of Silicon, Oxygen, and Calcium represent the Silica and Calcium silicate compounds.

### Section 3: In Vitro CalBot Cementation Experiments–Tooth Sample Preparation

Human teeth samples were acquired from human subjects, and twenty (n = 20) tooth samples were prepared for performing the in vitro experiments. These samples were obtained from Dr. Natasha Valijee and Dr. Prerna Krishna Modi, clinical residents at JSS (Jagadguru Sri Shivarathreeshwara) Dental College and Hospital, and were cleared by the JSS Dental College Institutional Ethics Committee (Approval Number: JSSDCH26/2020). The sample consisted of freshly extracted maxillary teeth for reasons including orthodontic correction, tooth impaction, and avulsed teeth due to traumatic accidents from age groups ranging from 20 to 55 years free of any cervical and carious lesions. The teeth samples were stored in 0.5% chloramine T‐hydrate (Sigma‐Aldrich, USA). Enamel defects on the tooth surface were created using a water‐cooled low‐speed IsoMet‐1000 annular saw (Buehler, USA) under a constant speed of 500 rpm. Following the creation of the enamel defects, the tooth was subjected to a cleaning process. The irrigants were used in the following sequence:

1. Ultrapure water (10 ml): Through rinse followed by air drying of the defect area.
2. 17% ethylenediaminetetraacetic acid gel at 6 pH (EDTA; Sigma‐Aldrich, USA)
3. Ultrapure water (10 ml) final rinse followed by air drying of the defect area.

Once the tooth samples were primed for the experiment, they were positioned in an artificial jaw to simulate clinical conditions followed by the treatment protocol. In both the groups, the respective CalBots solution was loaded on the defective tooth's surface and was driven using an array of magnets, which generated a gradient field of 1500G/cm along a direction perpendicular to the dentine enamel junction, facilitating the drive of CalBots towards the depths of exposed dentinal tubules in contrary to healthy dentinal tubules sealed by a layer of enamel. Teeth in both groups were subjected to treatment for 20 minutes, after which the samples were split along the enamel defect using a chisel and mallet. The samples were then loaded onto the SEM for analysis of the dentinal tubules corresponding to the enamel defect.

### Micro‐CT Analysis

After the usual Calbot treatment was done on the ex vivo teeth sample, the dentine disc was washed gently in water and then left in a concentrated 40 nm silver nanoparticle solution solution for 3 days. Micro‐CT was done using Xradia Versa 500 available at the Advanced Facility for Microscopy and Microanalysis, IISc Bangalore, with a sub‐micro voxel resolution of 0.7 µm. For this experiments, the source energy was kept at 140 kV, and the power was kept at 10 W. No source filter was used (Air), and a 5‐second exposure was used for scanning. 4x objective was used for imaging, which allowed the observation of approximately 750 µm by 750 µm by 750 µm volume. The total run time for one observation was approximately 3.5 hours.

To calculate the fast Fourier transform of every micro‐CT slice, the reconstructed stack was imported into Fiji (ImageJ^[^ ^48^ ^]^) as. tiff file. The stack was cropped to a 350 µm by 350 µm area and analyzed with the following macro:

 setBatchMode(true);

 stack = getImageID;

 n = nSlices;

 for (i = 1; i≤n; i++) {

selectImage(stack);

setSlice(i);

run(“FFT”);

}

run(“Images to Stack”, “name = [FFT Stack] title = FFT”);

setBatchMode(false);

To calculate the number of tubules from the contrast in the stack, the following logic was used: On Average for XRADIA 500 a single pixel corresponds to 0.7 micron

*Tubule diameter ranges from 0.75(lowest)(1.09px) to 3(highest)(4.28px) micron*

*In pixel square terms that need to look for areas in the range 1.2px^2^ to 18 px^2^*

With the above numbers in mind, the following macro was used to count the number of tubules in each slices. The threshold was chosen so that dentinal tubule contrast was selected:

setThreshold(LowerThreshold, UpperThreshold);

run(“Analyze Particles…”, “size = 1.2‐18 pixel summarize stack”);

After the count of open tubules was calculated from each micro‐CT slice, 50 subsequent stacks were averaged over to account for counting errors and any other artefacts that may arise due to thresholding.

### FFT Analysis

The radial averages of the FFT intensity peaks represent interpore distances in the Fourier space. For the FFT analysis to be done, the FFT units were first derived. This image was calibrated such that 1 pixel was approximately 0.75 microns. This gives the spatial resolution of the micro‐CT as 0.75 µm per pixel. The FFT image represents spatial frequency in cycles per unit distance. The frequency unit was the reciprocal of the spatial unit, which in this case was cycles per micron (µm⁻¹). The **Nyquist frequency** (the highest frequency that can be represented) was given by:

(1) $$f_{max}=\;\;\frac{1}{2\;\times pixel\;size}=\;\;\frac{1}{2\;\times 0.75}=\;0.67\;cycles/\mu m$$

This means the highest frequency visible in the FFT image was **0.67 cycles per micron**. The center of the FFT image represents the zero frequency (DC component). The edges of the FFT image correspond to the Nyquist frequency (0.67 cycles/µm). The distance from the center represents increasing spatial frequencies, measured in cycles per micron (µm⁻¹).

The relationship between distance in the FFT image and real‐world frequency was as follows:

(2) $$f\left(\frac{\mathrm{cycles}}{\mu m}\right)=\frac{distance\;from\;the\;center\;in\;FFT\;image}{image\;Width\;\left(in\;Pixels\right)\times pixel\;size\;in\;\mu m}$$

### Determine Frequency Per Pixel in the FFT Image

Since the FFT image was the same size as the original (350×350 px), the scale in the FFT image can ascertain:

(3) $$scaleX=\;\;\frac{f_{\max X}}{\frac{width}{2}}=\;\;\frac{0.67}{175}=0.0038\frac{cycles}{\mu m}perpixel$$

(4) $$scaleY=\;\;\frac{f_{\max Y}}{\frac{height}{2}}=\;\;\frac{0.67}{175}=0.0038\frac{cycles}{\mu m}perpixel$$

### Section 4: Randomized Control Trials in Mice Population

Animal trials were conducted in two distinct phases. Phase 1 trials encompassed the evaluation of the treatment protocol's (administration of CalBot suspension in CaO gel) efficacy in treating a cohort of disease‐induced mice population. Phase 2 trials included other control groups, aiming to establish the comparative effectiveness of the treatment protocol. All animal experiments were conducted with strict accordance to protocol approved by the Institutional Ethics Committee (IAEC) of the Indian Institute of Science (clearance from the Institutional ethics committee (CAF/ETHICS/128/2025)). In this study, a total of thirty‐two (n = 32) CD1 mice, comprising an equal number of males and females, aged between 8–12 weeks, and weighing 20–22 g, were utilized. For Phase 1 (n = 12), of the study, twelve mice were allocated equally into treated and non‐treated groups while Phase 2 (n = 20) involved twenty mice, equally distributed among four distinct control groups. The mice were assigned to their respective groups randomly. All mice were housed in plastic cages with a controlled temperature of 23 °C and a 12‐hour light/dark cycle. Commercial pellet diet and autoclaved water were provided ad libitum until a day before the experiment at which point the mice were water deprived. All mice underwent a sensitization procedure conducted under anesthesia to artificially induce DH. Following the sensitization protocol the mice were allowed to recover for a day by giving them a pellet diet and autoclaved water ad libitum.

### Sensitization Protocol

Mice were anesthetized with 2% isoflurane/oxygen before and during the surgery. The mice were head fixed in an inverted position on the stereotaxic to allow easy access to the teeth for the experimenter, as illustrated in Figure 5b for the ease of visualization of the operating field an operating microscope (3.5X zoom) was also installed. Using a water‐cooled handheld drill, the lower mandibular incisors were drilled until the enamel layer was breached and the dull yellow colored dentin was visibly exposed under the microscope, at which point the drilling was stopped. The mice were allowed to recover for a day by giving them a pellet diet and autoclaved water ad libitum.

### Behavioral Setup and Assay

The experimental setup (Figure 5a) comprises a plastic enclosure containing two syringes. One syringe was filled with water at ambient temperature, while the other has cold water. To ensure water deprivation, the mice were not provided water for one day before the commencement of the experiment. The mice were placed within the plastic cage, allowing them unrestricted movement to access water from either syringe. Additional water was dispensed upon the mouse licking from a syringe, allowing the mouse to continue drinking from the same syringe or switch to the other. Each trial session was standardized to a duration of 10 minutes. Trial session refers to a 10 minute duration during which the mice were placed in the behavioral setup and allowed to roam around to drink water either from the cold or the warm water syringe. The time limit for the trial sessions was standardized to 10 minutes per session based on preliminary experiments while testing the effectiveness of this behavioral assay. Licks were very rarely observed closer to the 10th minute mark as most mice stop licking around the 7th or 8th minute. From the 7th to 8th minute mark, mice start to show grooming and exploratory behavior indicating that they have quenched their thirst. All experimental procedures adhered to the protocols approved by the ethics committee at the Indian Institute of Science.

### Treatment Protocol for Animal Trials

Mice were anesthetized using a 2% isoflurane/oxygen mixture before and during the surgical procedure. The mice were securely positioned in a stereotaxic an implant apparatus, with their orientation inverted so that the experimenter had access to their teeth rather than the top of their skulls. In the treatment group of mice (n = 6), the treatment procedure involved the application of CalBots to the affected tooth surfaces. 1 mg of CalBot powder was uniformly suspended in a 0.1% w/v solution of calcium oxide (CaO) in distilled water, referred to as the CalBot solution. Before applying the CalBot solution, an array of magnets generating a magnetic gradient field of 1500 G/cm was affixed to the lingual side of the incisors in the mice. The 1500 G/cm gradient field was implemented by a permanent neodymium magnet of appropriate magnetization strength. It was experimentally confirmed that within the centimeter region where the teeth was subjected to this field, the gradient was almost constant. Subsequently, the CalBot solution was applied to the facial surface of the mandibular incisors, where enamel defects had been induced during the sensitization protocol. The magnet array was left undisturbed on the lingual surface of the incisors for 20 minutes to facilitate the formation of CalBot plugs within the dentinal tubules. In the non‐treatment group of mice (n = 6), the mice were also positioned in the stereotaxic setup and kept in place for 20 minutes to replicate conditions similar to those of the treatment group without introducing the CalBot solution.

### Justification for Stimulus Temperature

While Jafarzadeh & Abbott, *Int. Endod. J*., 201 0^[^ ^49^ ^]^ suggests that temperatures below 0 °C were classified as “cold” in human pulp sensibility tests, it was emphasize that the cold‐sensitive A‐fibers in teeth do not exclusively respond to subzero temperatures. Studies have shown that they respond to rapid drops in temperature, regardless of whether the final temperature reaches below 0 °C. (^[^ ^50^ ^]^ Bernal et al. (*Sci. Adv*., 2021^[^ ^51^ ^]^) identified the threshold for cold nociception in mice teeth to be around 19 °C, with rapid firing occurring due to a rapid temperature drop, rather than the absolute cold temperature. Based on these findings, the choice of 10.8 °C as the cold stimulus falls well within the range required to evoke sensitivity, making it biologically relevant for mice while mimicking real‐world cold exposure in daily life rather than extreme artificial conditions.

### Section 5: Statistical Analysis

All statistical analyses were performed using GraphPad Prism 7/9 software. ns>0.05, *≤0.05, **≤0.001, ***≤0.0001, ****<0.0001. For all experiments significance was established by One‐way ANOVA.

## Conflict of Interest

The authors declare no conflict of interest.

## Supporting information



Supporting Information

Supplementary Movie S1

Supplementary Movie S2

## Data Availability

The data that support the findings of this study are available in the supplementary material of this article.
