# Supplementary material for: Directed Self‐Assembly of Magnetic Bioceramic Deep Inside Dentinal Tubules May Alleviate Dental Hypersensitivity
Source: Adv Sci (Weinh). 2025 Jul 17;12(39):e07664. doi: 10.1002/advs.202507664 (PMC12533301; doi:10.1002/advs.202507664)
Supplement: Supplementary file 1 — Supporting Information [file ADVS-12-e07664-s002.docx]

**Supporting Information**

**S1: Magnetic manipulation and agglomeration of Calbots**

| **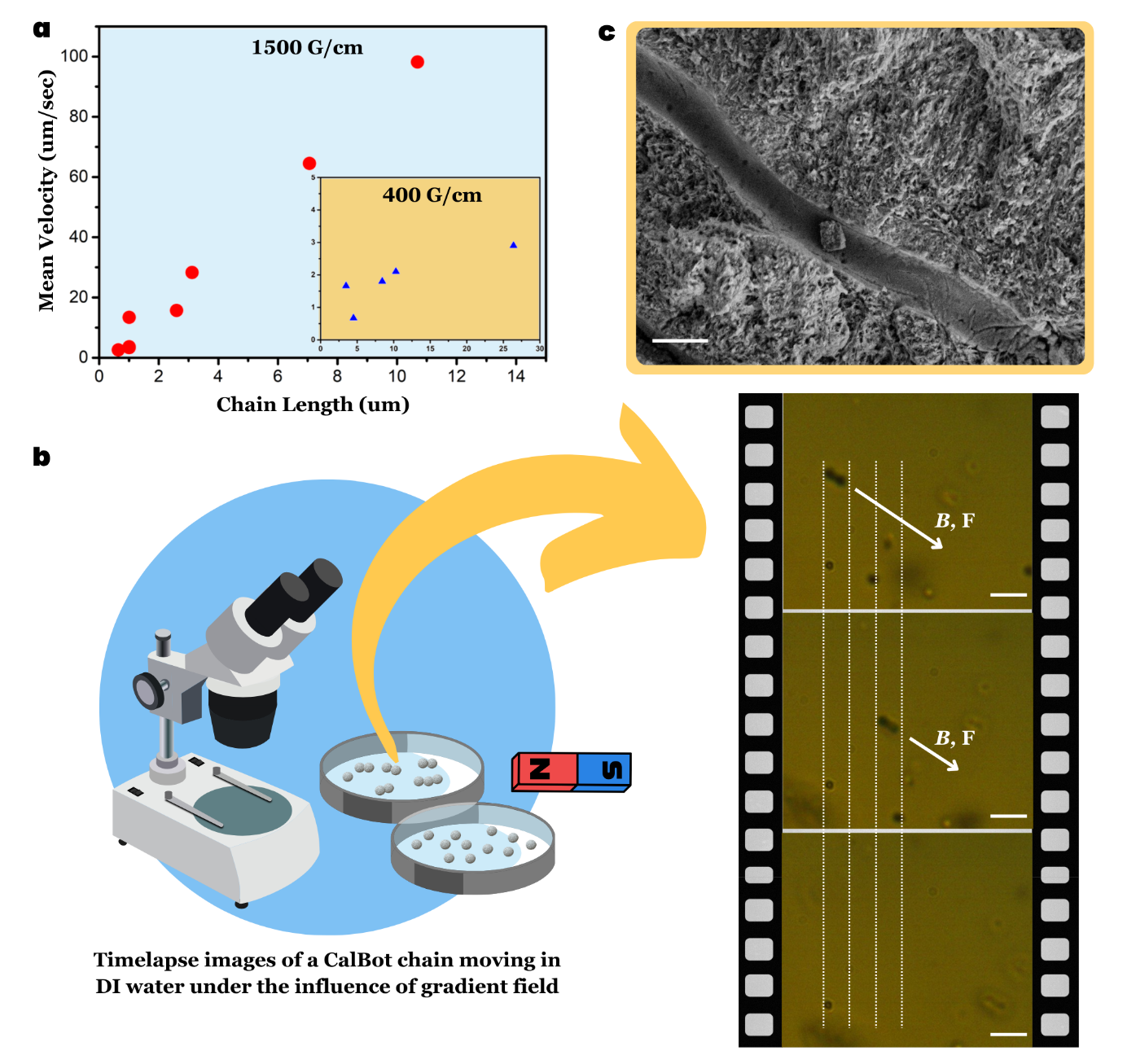**  **Figure S1** (a) Mean velocity measurement for different chain lengths at gradient magnetic field of 1500 G/cm and 400 G/cm (inset). (b) Timelapse images of a CalBot chain moving in DI water under a gradient field. Scale bar represents 5 μm (c) Representative image showing an incomplete plug formation at a location where the dentinal tubule curves away from the direction of motion. The scale bar represents 2 μm |
| --- |

As shown in Fig 2(a), a permanent magnet with a known gradient was used to study chain formation and velocity of CalBot chains in DeIonized (DI) water. The magnetic force $F_{mag}$ is given by $F_{mag}= \frac{1}{2}\frac{V\Delta\chi}{\mu_{o}} \nabla B^{2}$, where $\Delta\chi$ is the net magnetic susceptibility, $V$ is the volume of the beads, $\mu_{o}$ is the permeability of free space, and $B$ is the magnetic field ^1^. The drag force is given by $F_{d}=4\pi\eta av\frac{n^{2}-1}{\left( \frac{2n^{2}-1}{{(n^{2}-1)}^{1/2}} \right)\ln\left[ n+{(n^{2}-1)}^{1/2} \right]-n}$, where $a$ is the diameter of the sphere, $\eta$ is the viscosity of the medium, $u$ is the magnitude velocity of the object, and $n$ is the number of particles in the chain. The magnetophoretic velocity $u$ of a single CALBOT is determined through force balance and given by $v= \frac{V\Delta\chi}{4\pi\eta a}\frac{|\nabla B^{2}|}{2 \mu_{o}}\frac{\left( \frac{2n^{2}-1}{{(n^{2}-1)}^{1/2}} \right)\ln\left[ n+{(n^{2}-1)}^{1/2} \right]-n}{n^{2}-1}$. Our measurements of single CALBOTs velocities in DI water, as shown in Fig S1(a), provide us with an estimate of saturation magnetization, $m \sim2.5-5 \times{10}^{-14}Am^{2}$. We could observe the movement of CalBot chains in a small region of interest under the microscope where the gradient could be assumed linear, as shown in Fig S1(b). Our measurements of CALBOT chain velocities in DI water (See Figure S1(a) and (b)) show the formation of chains of length up to 10.6$\mu$m moving at a maximum velocity of up to 98.2 $\mu$m/s under a gradient magnetic field of 1500 G/cm, which is in agreement with previous work by Farraudo et al^2^.

As mentioned in the main text and empirically observed in multiple electron micrographs, the topography of the dentinal tubules plays an essential role in the depth of penetration of the CalBots. As shown in Fig S1(c), an incomplete plug formation at a location where the dentinal tubule curves away from the direction of motion could be observed. This sample was preserved for over 20 hours before sample preparation for electron microscopy.

In Fig 1(c), we confirm calcium silicate hydrate gel matrix formation when CalBots are suspended in CaO solution using a Transmission Electron Microscope (TEM).


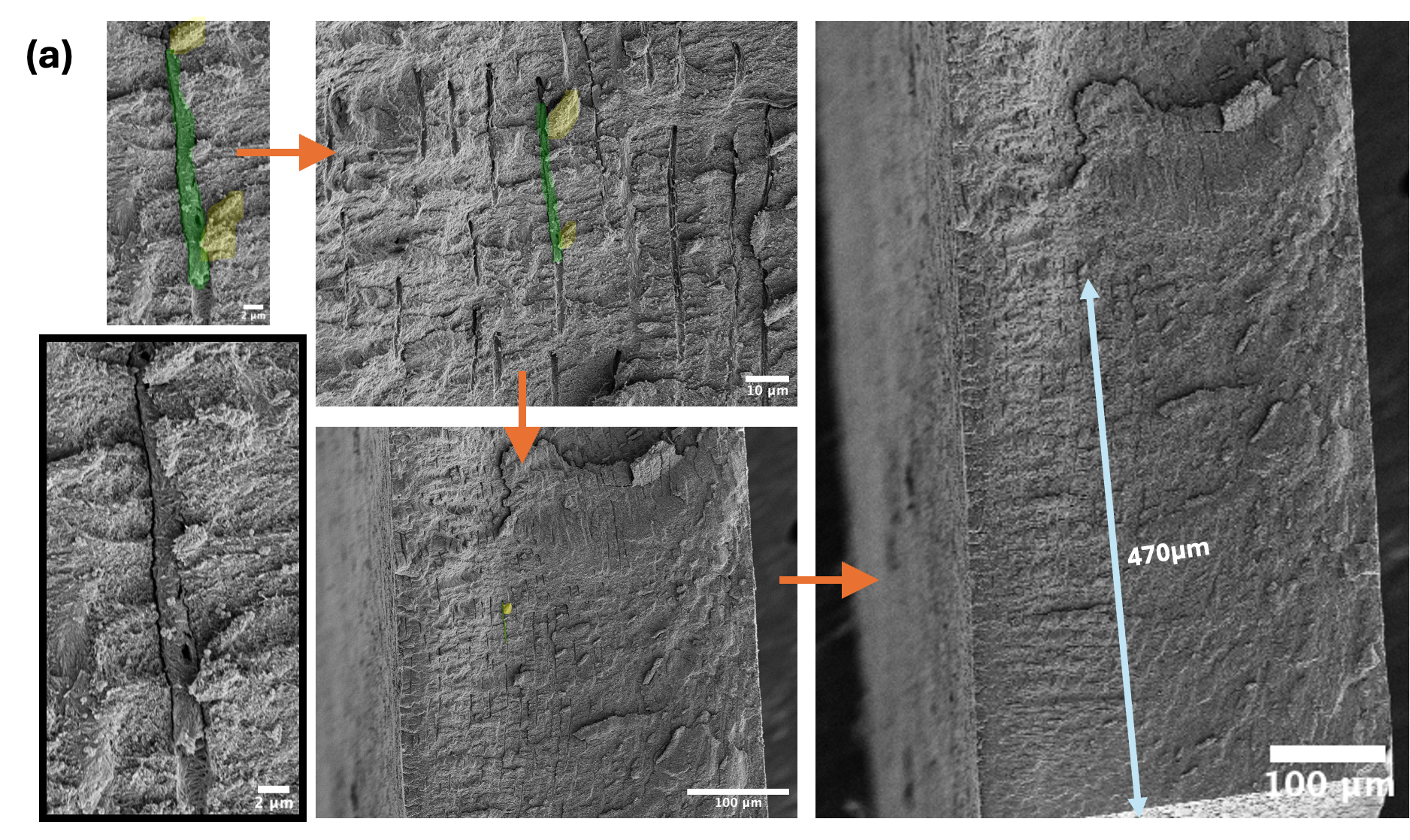


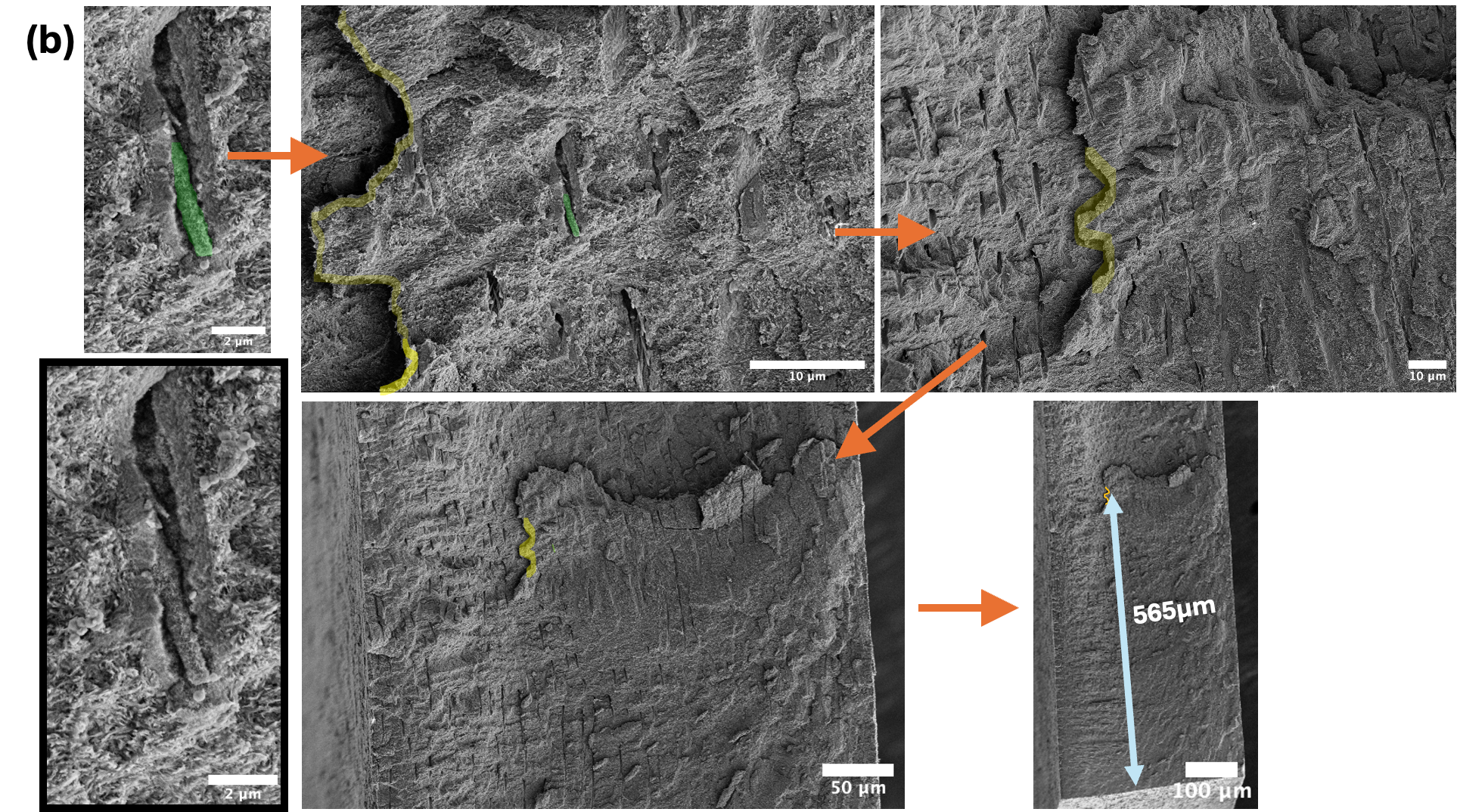


**Figure S2**: Scanning Electron Microscope images of plug formation taken at different magnifications showing the presence of plugs deep within the dentinal tubules. The maximum depth that could be observed was 565 microns

Due to the natural bend of the dentinal tubules, occlusion becomes discontinuous at greater depths. This does not imply there is only a single plug in the entirety of the tubule. In our experiments, we observed plug formations extending more than 500 µm as shown in Figure S2, which are inherently discontinuous due to the bending and branching of dentinal tubules at greater depths. This is consistent with the natural architecture of dentine, where tubules become less linear and more branched as they approach the pulp. Indeed, our CT images, animal studies and hydraulic conductance experiments show significant functional outcomes. This is expected since the fluid flow through a pipe can be stopped through multiple plugs formed at different points along the length of the pipe, as is the case here.

**S2: Air tightness assessment**

Darcy’s Law is usually applicable to measuring flows in porous systems.

For dentine, the Darcy’s law can be written as: $Q= A.K.\frac{\Delta p}{(\rho g)L}$

Where $Q$ is the volume flow rate of fluid measured in m^3^/s , $A$ is the cross-sectional area through which the fluid flows measured in m^2^, $K$ is the permeability of dentine measured in m/s and $\frac{\Delta p}{(\rho g)L}$ is the hydraulic gradient driving the flow where $\Delta p$ is the suction pressure created by the pump, $\rho$ is the density of the fluid flowing through the dentine (air), $g$is the acceleration due to gravity, and $L$ is the length of the dentine section.


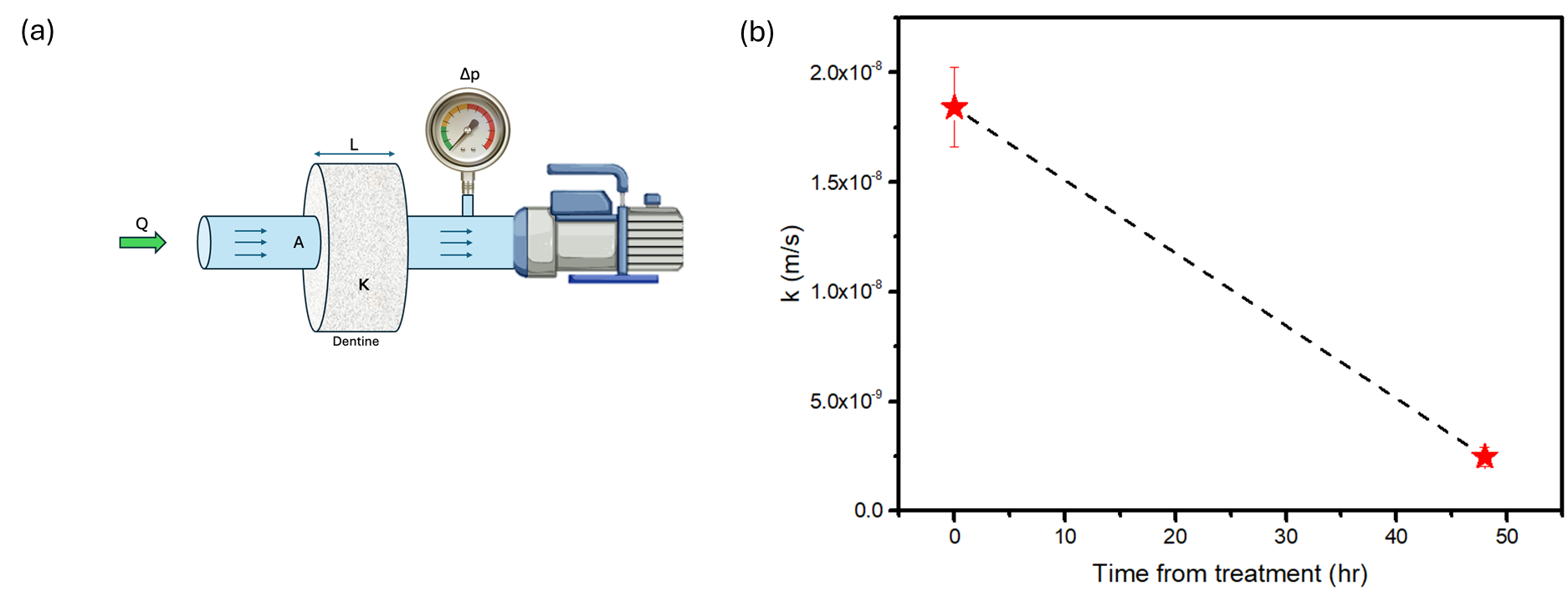


**Figure S3**: (a) Schematic showing the flow measurement system used to measure dentine permeability. (b) Permeability of dentine measured before treatment and 48 hours post-treatment. After 48 hours, we observed 87.7% reduction in permeability.

**S3: Sub-acute toxicity tests of CalBots in mice population**

In this paper, we perform animal trials as they serve as a critical bridge for translating preclinical research to human trials, ensuring the safety and efficacy of medical interventions. Before commencing animal trials, we investigated the sub-acute toxicity of CalBots in animal models. For our study, twenty (n=20) healthy five weeks old female BALB/c mice weighing 20-22g were acquired from Central Animal Facility (CAF), Indian Institute of Science (IISc), Bangalore, and acclimated for one week before the start of the experiment. Animals were housed in transparent polypropylene cages covered with securely fitted filter tops. Room temperature was maintained at (25± 2°C), relative humidity (40-50%) & a 12 h light/dark cycle. Distilled water & sterilized food for mice were provided *ad libitum* during the experimental period, as presented in Table 1.

| Groups | Route of administration | Test Dose | Test groups and Dose (mg/kg)  (200μl) | Adverse effect  (7 days) | Adverse effect (14, 28 days) | Number of mice |
| --- | --- | --- | --- | --- | --- | --- |
| 1 | i.p. | Single | 17.5 | None | None | 4 |
| 2 | i.p. | Single | 55 | None | None | 4 |
| 3 | i.p. | Single | 175 | None | None | 4 |
| 4 | i.p. | Single | 550 | None | None | 4 |
| 5 | i.p. | Single | PB S | None | None | 4 |

**Table 1**: Grouping of mice for the sub-acute toxicity tests. In all four test groups, no adverse effects of CalBots were observed after the observation period's 7^th^, 14^th,^ and 28^th^ days.

The toxicity tests were conducted as per Organization for Economic Cooperation and Development (OECD) guideline 425. All mouse groups were injected with their respective test dosage of CalBots ranging from 17.5mg/kg to 550mg/kg through the intraperitoneal route and kept under observation. After 14 days of observation, around 200-250μL of blood was collected using the retro-orbital method to analyze the complete blood count and serum parameters. Mice were sacrificed on day 28th for blood parameters and various organs as liver, spleen, kidney were collected for histopathological examination. No mortality was observed in any animal across all groups, and the mice did not display any sign of distress such as hunched posture, squinting of eyes, lack of appetite, lethargy, or discoloration of excretions. The body weight and Total blood count (TBC) of the various treatment groups were like that of the control group, and the serum values were within the reference range for Balb/c mouse, as illustrated in Table 2. In conclusion the results of this sub-acute toxicity study suggest that CalBots can be considered as safe for use as they do not cause any toxicity in mice population at a dose up to 550mg/kg.

| \|  \| Control \| 17.5mg/kg \| 55mg/kg \| 175mg/kg \| 550mg/kg \| Reference Range \| \| --- \| --- \| --- \| --- \| --- \| --- \| --- \| \| ALT (U/L) \| 44.1-55 \| 30.8-55.9 \| 28.9-72.2 \| 55.2-74.5 \| 20.4-55.2 \| 17-77 \| \| AST (U/L) \| 134-239 \| 220-229 \| 122-250 \| 172-225 \| 192-282 \| 54-298 \| \| ALP (U/L) \| 63.7-65.5 \| 84.3-95.1 \| 62.3-96 \| 56.6-72.8 \| 80.2-96 \| 35-96 \| \| Creatinine (mg/dl) \| 0.2 \| 0.1-0.6 \| 0.2 \| 0.2 \| 0.2 \| 0.2-0.9 \| \| Calcium(mg/dl) \| 6.6-10.5 \| 9.8-10.2 \| 9.1-10.6 \| 9.6-10.1 \| 9.4-9.8 \| 8-10.6 \|   **Table 2**: To test the toxicity of the CalBots, we injected them in Balb/c mice intraperitoneally for 28 days; CalBots were found to be non-toxic even to the extent of 550 mg/kg body weight, which was confirmed by the serum parameters as highlighted in the table. The Total Blood Count (TBC) values were also within the reference range for the test mice population and showed no toxicity during the entire experimental and observational period of 28 days. |
| --- | --- | --- | --- | --- | --- | --- | --- | --- | --- | --- | --- | --- | --- | --- | --- | --- | --- | --- | --- | --- | --- | --- | --- | --- | --- | --- | --- | --- | --- | --- | --- | --- | --- | --- | --- | --- | --- | --- | --- | --- | --- | --- |

**S4: Histopathology data analysis from the mice population for toxicity assessment**

Post 28^th^ day of the toxicity study, mice were euthanized by cervical dislocation, and organs were collected for histopathological grading across all treatment and control groups. The liver, kidney, and spleen were stored in PFA and further processed for H&E staining. The tissue samples were graded and assessed under 10X and 40X magnification.

| 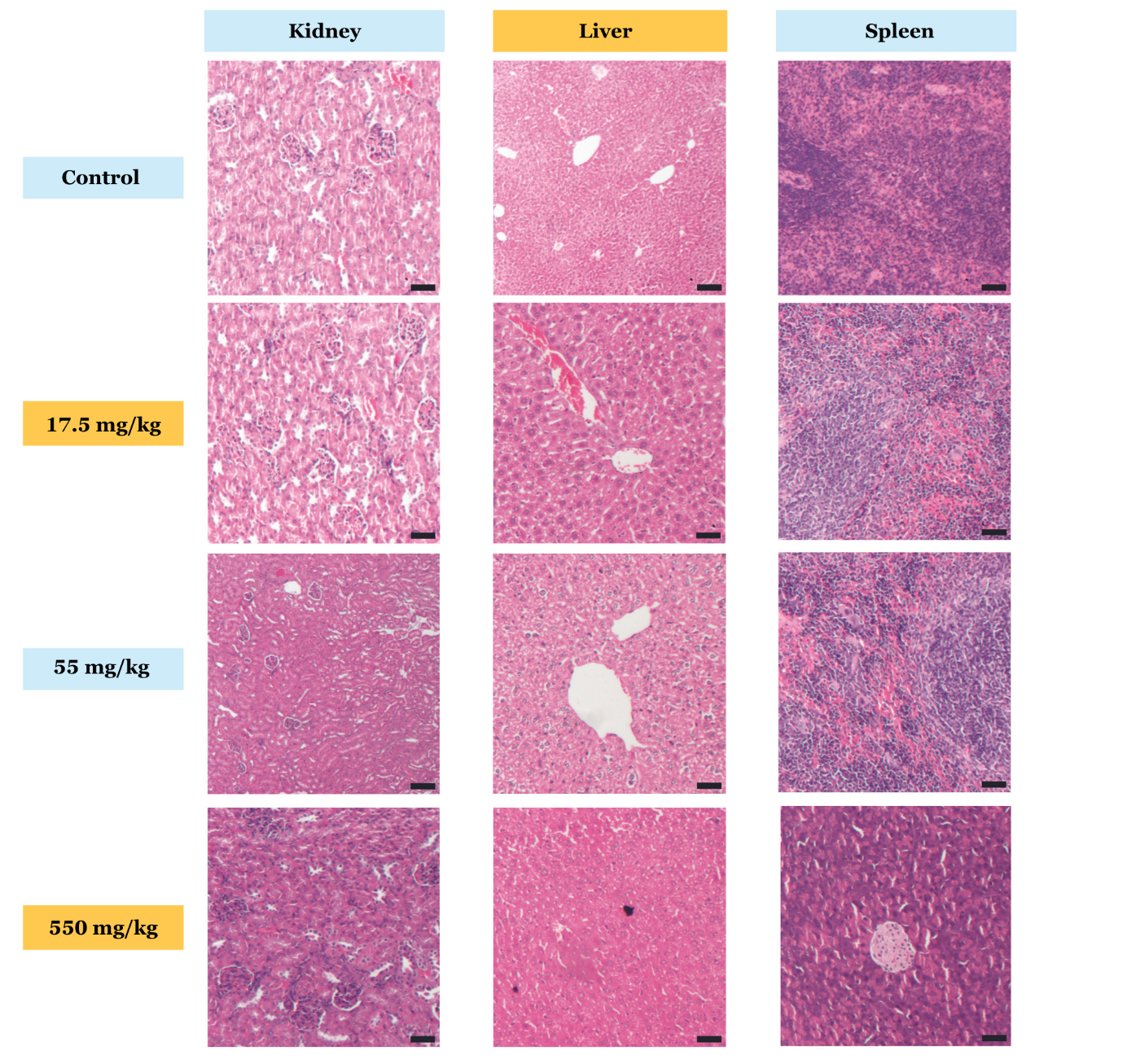 |
| --- |

**Figure S4**: Histopathology of the kidney, liver, and spleen of Balb/c mice of various treatment groups.

**Control**: The kidney section shows normal renal cortex and glomerular tufts. The liver section shows typical hepatic architecture with bi-nucleated cells. The spleen section shows typical splenic architecture and sinuses.**17.5mg/kg treated mice**: Kidney with healthy glomerulus and abundant capsular space. Liver with Normal architecture showing normal hepatocytes. Spleen with well-defined red and white pulp.**55mg/kg treated mice**: Renal arteriole with entrapped fragmented red blood cells. Liver with Normal architecture showing normal hepatocytes. Spleen with well-defined red and white pulp. **550mg/kg treated mice**: (a) Kidney sections with entrapped RBC (b) Liver sections with slight loss in architecture and ill-defined hepatocytes (c) Spleen with not very well defined red and white pulp, normal splenocytes

**Movie 1**: In the presented motion clip, we illustrate representative instances from controlled animal trials on a mice cohort, wherein discernible behavioural alterations were systematically tracked using an AI software DeepLabCut^©^ concerning their preference towards ambient or cold water. The footage encapsulates the behavioural nuances of a model mouse throughout the baseline study, followed by its responses after the induction of dental hypersensitivity. The final segment of the motion clip encapsulates the subject's behaviour after the administration of treatment via our CalBot treatment protocol.

**Statistical analysis**

All statistical analyses were performed using GraphPad Prism 7/9 software. ns>0.05, *≤0.05, **≤0.001, ***≤0.0001, ****<0.0001

**S5: Charachterization of Calbots**

**
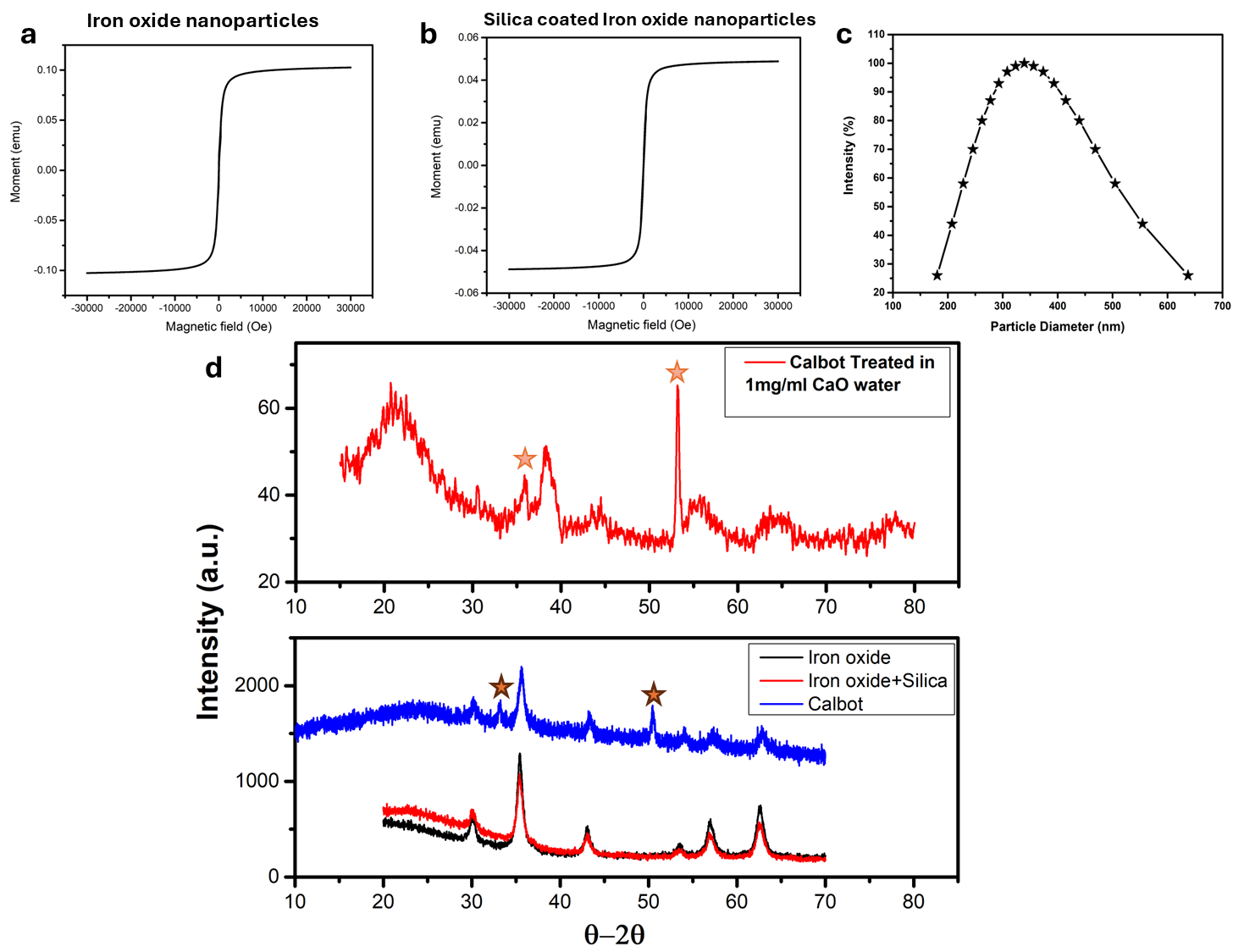
**

**Figure S5:** (a) Vibrating Sample Magnetometer (VSM) data analysis of iron oxide nanoparticles. (b) Vibrating Sample Magnetometer (VSM) data analysis of the silica coated iron oxide nanoparticle core-shell structure. (c) Probability distribution plot of particle size as obtained from dynamic light scattering experiments. (d) X-Ray Diffraction (XRD) Characterization of the different stages of Calbot synthesis as the particle goes through the stages of iron oxide nanoparticle to silica coating and finally the synthesis of Calbots. The upper graph shows XRD done after treating Calbot with 1mg/ml CaO-water for 10 minutes, before washing and subsequent drying for sample preparation.

Iron oxide XRD shows sharp peaks at 30°, 35°, 43°, 53°, 57° and 63° confirming the phase to be magnetite. During formation of the thin silica shell on iron-oxide core the sharp crystalline peaks of iron-oxide still dominate but their intensity is reduced. An amorphous signal from silica can be observed in the 20 - 30° region. Since the thin silica coating is amorphous, we did not oberve any new peaks.

To make Calbots, Calcium is introduced into the silica-coated iron oxide by annealing 10 mL silica-coated iron oxide nanoparticles ethanol solution in 0.2-0.4 gm of calcium nitrate tetrahydrate for 2-5 hours at 600°C. As observed in Figure S5 (d), after introduction of calcium, new peaks appear at 33.2° and 50.4° marked by a star symbol in the graph.

Calcium silicate-based materials such as tricalcium silicate and dicalcium silicate show strong diffraction peaks in the 30-34° range as well as reflections in the 50-55° range. The new peaks emerging at around 33° and around 50° can be attributed to tricalcium silicate as reported in multiple literature^3–5^. Please note, an alternate explanation is provided by Grangeon et. al. ^6^, who reports that jennite, a calcium silicate hydrate mineral, has maxima at ~50.7° and the XRD patterns hold true for disordered variations. Jennite is known to be formed during the hydration of Portland Cement. ^7^

The upper graph of Figure S5 (d) shows the XRD pattern after Calbot particles were immersed in 1mg/ml CaO-water solution for 10 minutes, to replicate the exposure of Calbot to CaO solution during entry into the dentinal tubules. After 10 minutes, the Calbot was washed and allowed to stay in deionized water solution for 24 hours. The XRD pattern appears slightly shifted, while the signature peaks are still recognizable. The iron-oxide peaks at 35° and 43° are still observed although they are now shifted to around 37° and 44.5° respectively. Overall it is evident that an amorphous coating reduces the intensity and broadens most of the crystalline peaks after reaction with CaO solution. The 50.4° is shifted to 53.6° but has become significantly more prominent compared to the 35.9° peak (shifted from 33.2°). This leads to believe the formation of new calcium silicate hydrate minerals at the final stage of the reaction. The XRD pattern does show a general increase in amorphous coatings which might indicate deposition of calcium hydroxide and continual formation of calcium silicate hydrate leading to plug formations in the dentinal tubules.

The XRD patterns and all the material characterization in the paper, lead us to believe that the plugs made by the Calbots have silica and various phases of calcium silicate, calcium silicate hydrate on their shell.

We believe the material composition, as per Solonenko et.al^8^., is due to exposed silica reacting with calcium hydroxide to produce calcium silicate hydrates:

$$rCa\left( OH \right)_{2}+m SiO_{2} +zH_{2}O\to r CaO.m SiO_{2} . n H_{2}O$$

For tricalcium silicates or other such silicates present on the Calbot shell, a general hydration reaction might occur leading to production of more calcium silicate hydrates:

$$2Ca_{3}\left( SiO_{4} \right)O+7H_{2}O\to3 CaO.2 SiO_{2} . 4 H_{2}O+3 Ca\left( OH \right)_{2}$$

Under ambient $CO_{2}$ some CSH converts to calcium carbonate as follows:

$$r CaO.m SiO_{2} . n H_{2}O+r CO_{2}\to r CaCO_{3}+m SiO_{2}+n H_{2}O$$

This has been ascertained by the TEM of calcium silicate hydrate crystals, indicating the presence of carbon where new amorphous growth was observed.


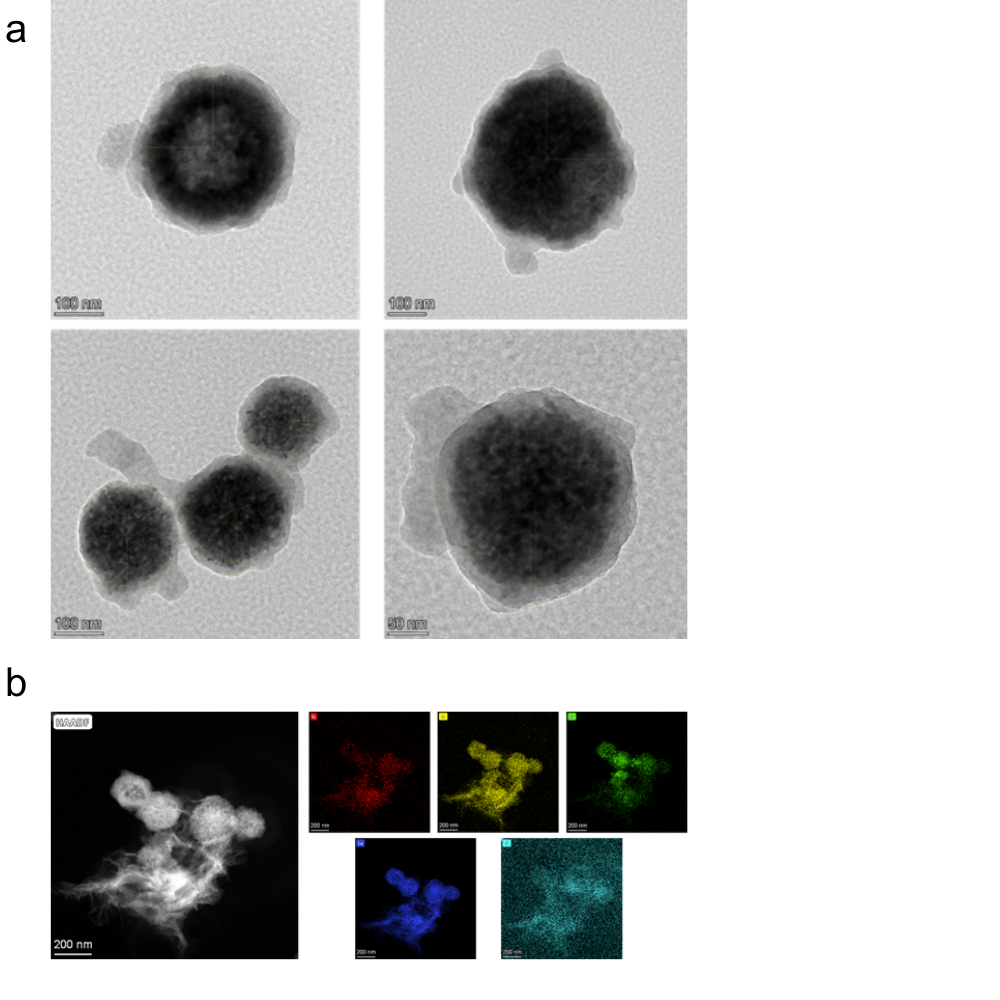


**Figure S6:** (a)Additional Transmission Electron Microscopy images of Calbots demonstrating a reasonable control over shape and size during synthesis. (b) Elemental composition after CalBot is kept in CaO solution. The colors for elements are as follows: Red-Silicon, Yellow-Oxygen, Green-Calcium, Blue-Iron and Cyan-Carbon

1. Zborowski, M., Fuh, C. B., Sun, L., Chalmers, J. J. & Green, R. Analytical Magnetapheresis of Ferritin-Labeled Lymphocytes. *Anal Chem* **67**, 3702–3712 (1995).

2. Faraudo, J., Andreu, J. S., Calero, C. & Camacho, J. Predicting the Self‐Assembly of Superparamagnetic Colloids under Magnetic Fields. *Adv Funct Mater* **26**, 3837–3858 (2016).

3. Mutluay, A., Mutluay, M. & Pehlivanli, A. Hydration reaction analysis of calcium-silicatebased materials using scanning electron microscopy and X-ray diffraction method. *Balkan Journal of Dental Medicine* **26**, 133–141 (2022).

4. Neelakantan, P., Berger, T., Primus, C., Shemesh, H. & Wesselink, P. R. Acidic and alkaline chemicals’ influence on a tricalcium silicate‐based dental biomaterial. *J Biomed Mater Res B Appl Biomater* **107**, 377–387 (2019).

5. Ren, X., Zhang, W. & Ye, J. FTIR study on the polymorphic structure of tricalcium silicate. *Cem Concr Res* **99**, 129–136 (2017).

6. Grangeon, S., Claret, F., Linard, Y. & Chiaberge, C. X-ray diffraction: a powerful tool to probe and understand the structure of nanocrystalline calcium silicate hydrates. *Acta Crystallogr B Struct Sci Cryst Eng Mater* **69**, 465–473 (2013).

7. Yu, P. & Kirkpatrick, J. R. Thermal dehydration of tobermorite and jennite. *Concrete Science and Engineering* **1**, (1999).

8. Solonenko, A. P., Blesman, A. I. & Polonyankin, D. A. Poorly crystallized hydroxyapatite and calcium silicate hydrate composites: Synthesis, characterization and soaking in simulated body fluid. *Mater Charact* **161**, (2020).
